# Integrated molecular and ADME-toxicity profiling identifies PGV-5 and HGV-5 as potential agents to counteract multidrug-resistant (MDR) cancer

**DOI:** 10.1038/s41598-025-02858-3

**Published:** 2025-10-21

**Authors:** Ritmaleni, Rosalina Diani Prima Anargya, Najla Alivia, Prajona Marbun, Retno Murwanti, Ranti Kamila Habibie

**Affiliations:** 1 Department of Pharmaceutical Chemistry, Faculty of Pharmacy, Universitas Gadjah Mada, Sleman, Yogyakarta 55281 Indonesia; 2 Curcumin Research Center, Faculty of Pharmacy, Universitas Gadjah Mada, Sleman, Yogyakarta 55281 Indonesia; 3 Departement of Pharmacology and Clinical Pharmacy, Faculty of Pharmacy, Universitas Gadjah Mada, Sleman, Yogyakarta 55281 Indonesia; 4 Faculty of Pharmacy, Universitas Gadjah Mada, Sleman, Yogyakarta 55281 Indonesia

**Keywords:** Curcumin analogue, Acute toxicity study, Histopathological examination, *In silico*-ADMET, Molecular docking, Network pharmacology, Chemistry, Medicinal chemistry, Computational chemistry

## Abstract

Curcumin, a pharmacological agent found in turmeric’s rhizome, has been studied for its various therapeutic properties. However, its clinical development is hindered by its instability and low solubility in water, resulting in inadequate oral bioavailability. Two potential curcumin analogs, 2,5-bis(4’-hydroxy-3’,5’-dimethoxybenylidene)cyclopentanone (PGV-5) and 2,6-bis(4’-hydroxy-3’,5’-dimethoxybenylidene)cyclohexanone (HGV-5), are being developed to address this issue and enhance their therapeutic efficacy. The study aims to screen novel curcumin analog compounds by integrating in silico assessment of ADME properties, acute toxicity studies, and computational analysis. PGV-5 and HGV-5 are classified as Global Harmonized System of Classification and Labeling of Chemicals (GHS) class 4 and class 5, respectively, in acute toxicity assessment, as they cause histopathological changes in the heart and lungs. Their ADME profile indicates they serve as effective P-glycoprotein (P-gp) inhibitors, making them potential candidates for development as anti-multidrug resistance agents, particularly in cancer cells. Molecular docking on P-gp revealed significant inhibitory capability relative to curcumin, exhibiting comparable binding characteristics to the native ligand, as evidenced by superior docking scores. Subsequent molecular dynamics simulations confirmed the stable interaction of both compounds with P-gp, with HGV-5 showing the most favorable binding free energy. Target gene mapping revealed several pivotal targets including AKT1, STAT3, EGFR, and NF-κB1. These findings suggest that PGV-5 and HGV-5 merit further research as agents against multidrug-resistant in cancer, regardless of their toxicity profiles. Further confirmation of their effects requires more laboratory studies and clinical trials.

**Supplementary Information:**

The online version contains supplementary material available at 10.1038/s41598-025-02858-3.

## Introduction

Multidrug resistance (MDR) is a major challenge in treating disease, particularly in oncology, where cancer cells are resistant to various therapies^1^. Overexpression of membrane transporter proteins, such as ABC transporters, leads to the expulsion of cytotoxic agents, diminishing therapy efficiency^2^. P-glycoprotein (P-gp) is a transmembrane protein which the overexpression of P-gp in cancer cells results in MDR^3^. Several products have been developed to modulate MDR by P-gp inhibition, but these require high dosages and cause toxicity issues^4,5^. An efficacious anti-MDR cancer treatment using natural compounds like curcumin has been developed.

Turmeric rhizome (*Curcuma longa* L., Zingiberaceae) a traditional medicine plant, has numerous pharmacological effects, including antioxidant, anti-inflammatory, antiparasitic, antimutagenic, anticancer, chemoprotective, antibacterial, and antiviral properties^6,7^. Curcumin (1,7-bis(4’-hydroxy-3’-methoxyphenyl)-1,6-heptadiene-3,5-dione), a yellow pigment derived from the rhizome of turmeric, is known for its antioxidant, anticancer, anti-inflammatory, and anti-hepatotoxic properties^8^. Curcumin, can suppress P-gp activity in the gut and multidrug-resistant cancer cells by altering its pharmacokinetic properties^9,10^. However, its clinical development is hindered by its instability and low solubility in water, leading to inadequate oral bioavailability and minimum effective therapeutic concentration^11,12^. This instability is due to its unstable structure, particularly the active methylene group from the diketone moiety, which leads to fast breakdown and metabolism^13^. Consequently, numerous studies have been conducted to address curcumin instability and improve its stability and pharmacological efficacy.

While extensive studies on monocarbonyl analogs have predominantly focused on their anticancer, antibacterial, anti-inflammatory, and antioxidant activities, their capacity to inhibit ABC transporters, particularly P-glycoprotein (P-gp), remains insufficiently explored. Notably, 23 heterocyclic cyclohexanone monocarbonyl analogs of curcumin were synthesized and assessed for their ability to inhibit ABC transporters, including P-gp, using flow cytometry and resistance reversal assays. Many of these compounds demonstrated greater chemical stability and more potent P-gp inhibitory activity compared to curcumin, with several outperforming curcumin in efficacy. Furthermore, some analogs exhibited notable anticancer properties, indicating their potential as dual-function antitumor agents, similar to curcumin’s mechanism. Additionally, 12 unsymmetrical curcuminoids with various amide groups were synthesized and tested for their ability to reverse multidrug resistance (MDR) in P-gp overexpressing MDR cervical adenocarcinoma cells, in combination with vincristine and paclitaxel, using verapamil as a positive control. Three compounds showed strong MDR reversal by effectively inhibiting P-gp-mediated drug efflux, while others demonstrated moderate activity. Preliminary structure–activity relationship analysis also revealed that only one-half of curcumin’s symmetrical structure, particularly featuring one or two chloride groups at the meta- or para-position of the benzamide moiety, serves as a promising lead for MDR reversal agents^14,15^.

**Fig. 1:**
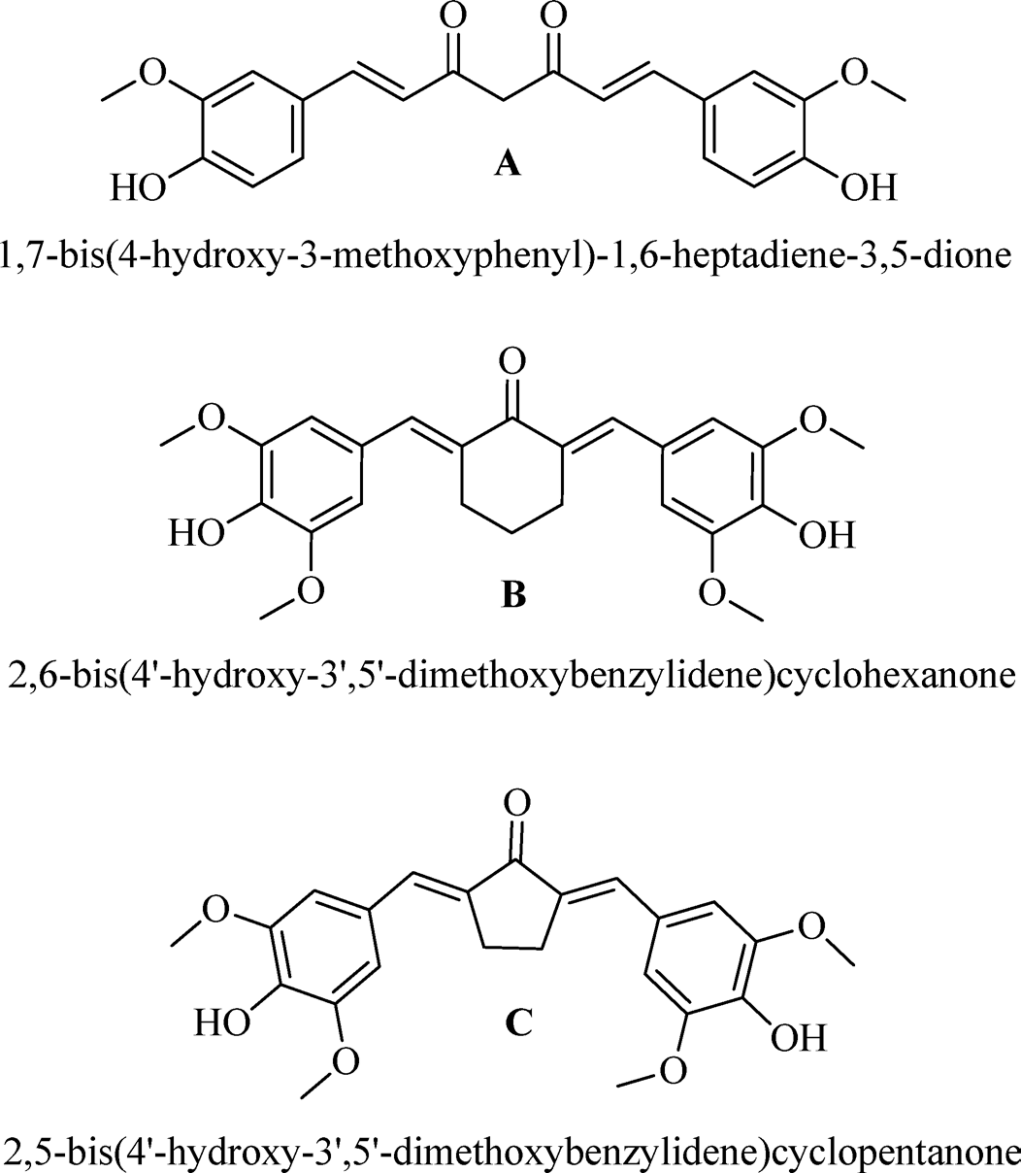
Chemical structure of (**A**) Curcumin, (**B**) HGV-5, and (**C**) PGV-5.

2,5-bis(4’-hydroxy-3’,5’-dimethoxybenylidene)cyclopentanone (PGV-5) and 2,6-bis(4’-hydroxy-3’,5’-dimethoxybenylidene)cyclohexanone (HGV-5) are monocarbonyl curcumin analogs previously synthesized by Sardjiman, patented (Patent Number: US677447B2), (Fig. 1) and studied for their anti-inflammatory, antibacterial, antifungal, and antioxidant properties^16^. These compounds undergo structural alteration by substituting the β-diketone core of curcumin with a cyclopentanone core (PGV-5) and a cyclohexanone core (HGV-5) which is expected to improve its stability and efficacy. Structure-activity relationship studies suggest that incorporating a methoxy group on the phenyl ring can augment the anti-inflammatory and antioxidant properties^17^.

**Fig. 2:**
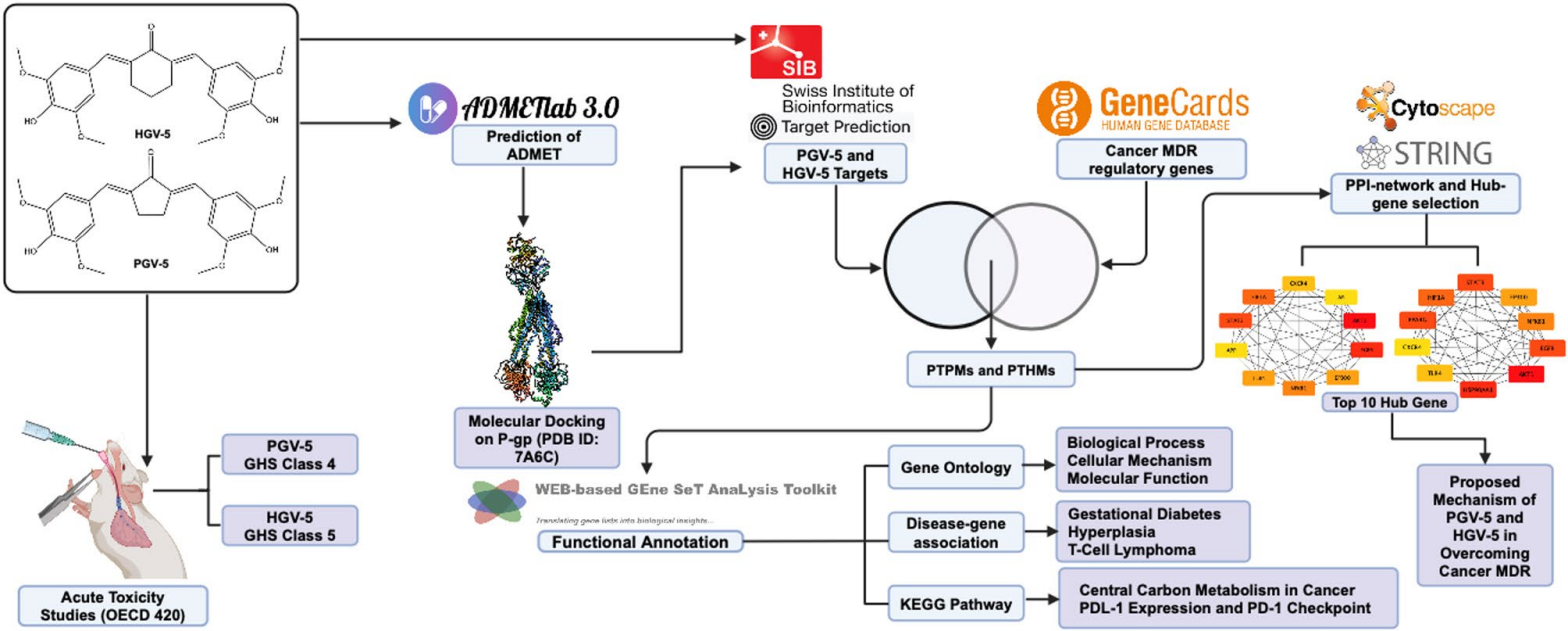
Work flow of study. Integrating in silico and in vivo studies for safety and activity profiling of curcumin analogues.

Pharmacokinetic profile assessment is a crucial stage in drug discovery and development, assessing the behavior of compounds in biological systems. ADMETLab 3.0 platform is utilized for this screening^18^ Oral acute toxicity tests assess the hazardous effects of short-term exposure, while the LD50 value aids classification according to the Globally Harmonized System of Classification and Labelling of Chemicals (GHS). Histopathological analysis of essential organs assesses the tissue-level biological effects^19^. Molecular docking is conducted to elucidate drugs-proteins interaction, identifying the crucial amino acid residues that underpin pharmacological activity^20^. This research combines bioinformatics analysis with network pharmacology to identify promising target hub genes for PGV-5 and HGV-5 in cancer multidrug resistance, improving understanding of curcumin analogs’ potential and predicting their development in cancer treatment resistance mechanisms (Fig. 2).

## Materials and methods

### Prediction of ADMET by computational analysis

The pharmacokinetic properties of PGV-5 and HGV-5 were analyzed using the ADMETLab 3.0 evaluation server (https://admetlab3.scbdd.com/server/evaluation)^18^ with the SMILES code entered into the website for preliminary screening.

### Chemical and reagents

The curcumin analog compounds PGV-5 and HGV-5 were obtained from Prof. Ritmaleni’s Laboratory of Curcumin Research Center (CRC), Faculty of Pharmacy, Universitas Gadjah Mada, Indonesia, following the synthesis procedure outlined by Sardjiman^16^. CMC-Na was purchased from Sigma (USA). Neutral Buffer Formalin 10% was purchased from Bio Optica Milano spa (Italy). Both compounds were prepared for acute toxicity testing on the same day to ensure stability and prevent deterioration.

### Experimental animals

The in vivo test protocol was carried out in accordance with relevance guideline and regulation that have been approved by the Research Ethics Committee of the Faculty of Veterinary Medicine, Universitas Gadjah Mada, Indonesia (Number: 54/EC/-FKH/int./2024). Furthermore, the study has been conducted and reported in compliance with the ARRIVE guidelines to ensure transparency and reproducibility. The test involved female BALB/C strain mice, aged 8–12 weeks with a weight of 20–30 g, obtained from the Department of Pharmacology and Toxicology, Faculty of Pharmacy, Universitas Gadjah Mada, Indonesia. The mice were fed Citra Feed RatBio Food (Indonesia) and drinking reverse osmosis water, ad libitum. The mice were maintained in suitable conditions with temperature ranges of 22 °C (± 3 °C), relative humidity between 30% and 70%, and 12 h of light followed by 12 h of darkness.

### Acute toxicity studies

Acute toxicity tests were conducted according to the OECD Guideline for Testing of Chemicals. (No. 420, Section 4, Health Effects)^21^. The study involved 3 different groups of mice. Groups 1 and 2 received PGV-5 and HGV-5 curcumin analogs in 0.7% CMC-Na, respectively. Group 3, as the solvent control group, was given 0.7% CMC-Na. A preliminary test was conducted. Post-treatment, the mice were monitored for signs of toxicity for 3–4 days. If no symptoms were observed, four additional test subjects were added to each group for the primary test. The animals were examined and weighed over a 14-day period for clinical changes, signs of toxicity, and mortality. All animals that died during and after the study were euthanized through neck dislocation, and the liver, spleen, heart, kidneys, and lungs were macroscopically examined and their relative weight calculated using a formula.

$${\text{Percent Relative Organ Weight }}=\frac{{{\text{Organ~weight~}}\left( {\text{g}} \right)}}{{{\text{Body~weight~of~rat~on~the~day~of~sacrifice~}}\left( {\text{g}} \right)}} \times 100\% $$

### Histopathological examination

Test animals were sacrificed with neck dislocation and the liver, spleen, heart, kidneys, and lungs were preserved in 10% Neutral Buffered Formalin (NBF) for 24 h. All organs were sectioned and dehydrated, then embedded in paraffin wax. The tissue was stained using hematoxylin and eosin (H&E) and examined under a light microscope Olympus BX53 at a magnification of 400x. A qualitative analysis was performed by observing morphological alterations in each organ relative to its solvent control (CMC-Na 0.7%).

### Molecular docking on P-glycoprotein

Molecular docking analysis was performed using Molecular Operating Environment (MOE) version 2015 (licensed under Department of Pharmaceutical Chemistry, Faculty of Pharmacy, Universitas Gadjah Mada)^22^. The structure of the P-gp (PDB ID: 7A6C) was downloaded from the RCSB website (https://www.rcsb.org) in PDB file format^23^. The protein was prepared by removing water molecules, solvents, and cholesterol, then adding hydrogen molecules using the “Protonate 3D” feature. Energy minimization was performed using the “Energy Minimization” feature in MOE with default settings. The docking method was validated by determining the binding site using the “Surface and Maps” feature and redocking. The results were considered valid if the RMSD value was less than 2 Å. The 2D structures of the PGV-5, HGV-5, and curcumin (as comparator) were converted into SMILES code and transformed into 3D structures using the “Builder” feature. The compounds were created into a database in MOE and prepared using charges using the “Partial Charge” and “Energy Minimization” features. The docking simulation used the site setting “Ligand Atoms” with placement “Triangle Matcher” and refinement “Induce Fit”. The scoring parameters used were “London dG” as scoring parameter 1 and “GBVI/WSA dG” as scoring parameter 2. The conformation of the structure with the lowest binding affinity value was analyzed and visualized in 2D and 3D.

### Molecular Dynamics Simulation and MM-PBSA

Molecular dynamics (MD) simulations were conducted using GROMACS 2023 with the CHARMM36 force field. Ligand topologies were generated via Avogadro and CGenFF, while the protein structure was prepared using pdb2gmx with CHARMM36 compatibility. The protein–ligand complex was solvated in a TIP3P water cubic box (1.2 nm margin) under periodic boundary conditions (PBC), and neutralized with Na⁺ and Cl⁻ ions. Energy minimization was performed using the steepest descent algorithm (100,000 steps) with Particle Mesh Ewald (PME) for electrostatics and EnerPres for dispersion correction (DipsCorr) for van der Waals interactions, both at a 1.2 nm cutoff.The system was equilibrated in two phases: NVT (Berendsen thermostat, 300 K) and NPT (Parrinello–Rahman barostat, 1 bar), each for 100 ps with a 2 fs time step, employing position restraints (1000 kJ mol⁻¹ nm⁻²) on the ligand. The production run was executed for 200 ns without restraints, maintaining the same parameters. Trajectory snapshots were saved every 10 ps for subsequent analyses including root mean square deviation (RMSD), the root mean square fluctuation (RMSF), radius of gyration (Rg), solvent-accessible surface area (SASA), and hydrogen bonding using GROMACS tools.Binding free energy calculations were performed using the Poisson-Boltzmann surface area (MM-PBSA) approach implemented in the gmxMMPBSA program. The total binding energy was estimated by summing vacuum molecular mechanics energy (van der Waals and electrostatic), polar solvation energy, and non-polar solvation energy. A total of 7500 frames, extracted every 10 ps from the 75–150 ns segment of the MD trajectory, were used for analysis. Additionally, per-residue decomposition was conducted to determine the specific energetic contributions of individual amino acids to ligand binding, providing detailed insight into key interaction sites.

### Network pharmacology analysis

The study used various databases to compile target proteins form PGV-5 and HGV-5 including SwissTargetPrediction (http://www.swisstargetprediction.ch) and Super Pred Target Prediction (https://prediction.charite.de/subpages/target_prediction.php) and GeneCards (https://www.genecards.org/#) to identify genes that modulate cancer multidrug resistance. InteractiVenn (https://www.interactivenn.net) was used to analyze overlapping genes from both targets fishing results, resulting potential targets of PGV-5 in cancer multidrug resistance (PTPMs) and potential targets of HGV-5 in cancer multidrug resistance (PTHMs)^24–27^. STRING version 11.5 (https://string-db.org) was used for protein-protein interaction analysis^28^. Cytoscape version 3.9.1 (https://cytoscape.org) was used to determine the top 10 genes using the Cytohubba plugin, employing the “Degree Score” parameter^29^.

The supplementary enrichment data for the hub gene included gene ontology analysis, KEGG pathway, and disease-gene association enrichment conducted via the WEB-based Gene SeT AnaLysis Toolkit (Web-Gestalt) website (https://webgestalt.org/)^30^. The study used the parameter method of interest “Over-Representation Analysis (ORA)” with the organism “Homo sapiens” and the functional database “gene ontology”. For disease-gene analysis, identical parameters were employed, and “DisGenet” was chosen as the functional database. KEGG pathways enrichment analysis was also conducted using the same parameter as the gene ontology analysis. Both FDR < 0.05 was employed as the threshold criterion.

### Statistical analysis

Quantitative data are expressed as mean ± standard error of the mean (SEM). All quantitative data from the acute toxicity test result were evaluated utilizing GraphPad Prism version 9.2.0. The Shapiro-Wilk method was employed to conduct the normality test at a 95% confidence level. The independent sample T-Test was used to assess statistical significance, with a *p*-value of < 0.05.

## Results and discussion

### Prediction of ADMET properties

**Fig. 3:**
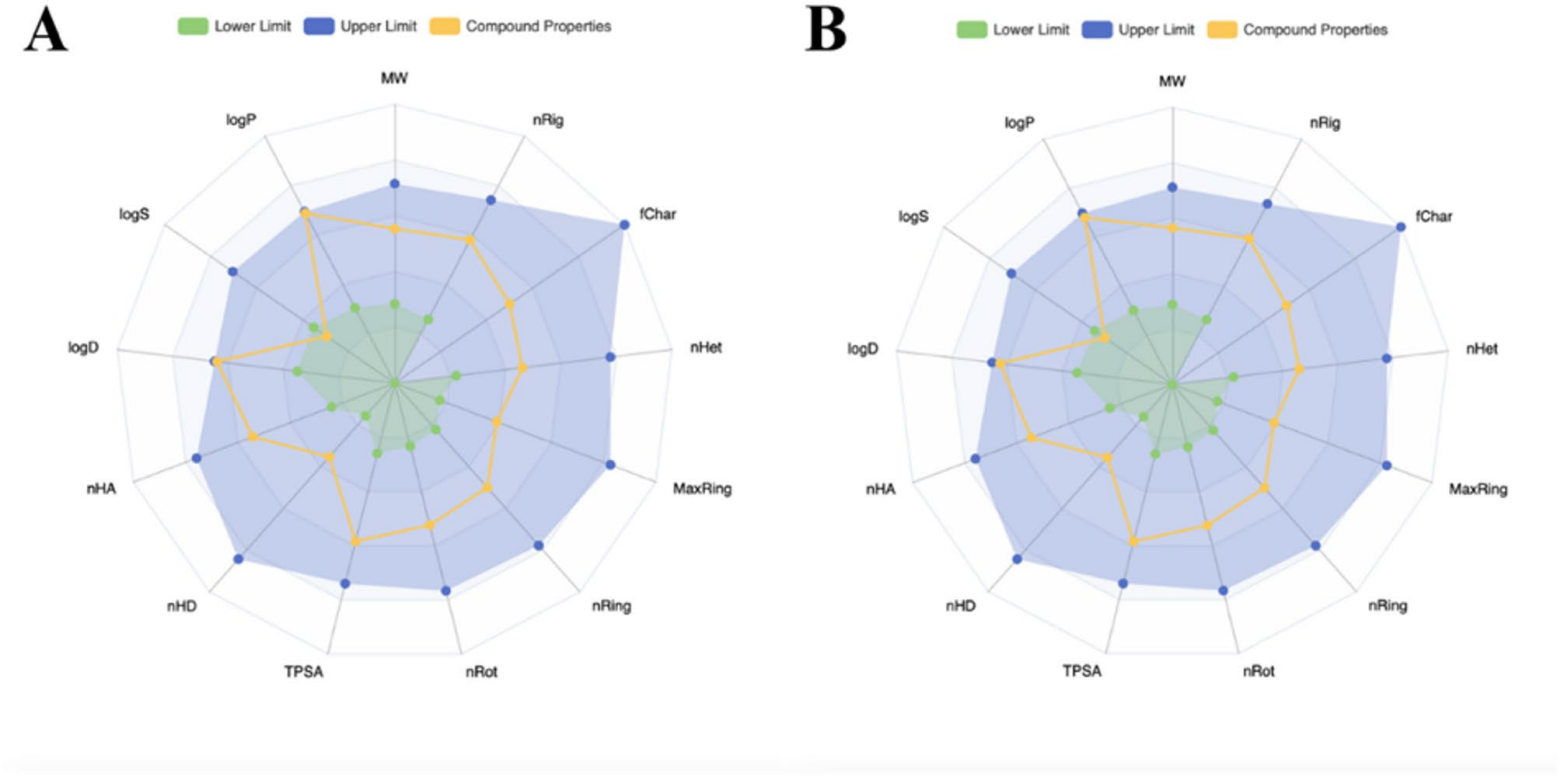
Physicochemical radar of PGV-5 (**A**) and HGV-5 (**B**) obtained from ADMETLab 3.0 web server. MW, molecular weight; nRig, number of rigid bonds; fChar, formal charge; nHet, number of heteroatoms; MaxRing, number of atoms in the biggest ring; nRing, number of rings; nRot, number of rotable bonds; TPSA, topological polar surface area; nHD, number of hydrogen bond donors; nHA, number of hydrogen bond acceptors; LogD, logP at physiological pH 7.4; LogS, log of the aqueous solubility; LogP, log of the octanol/water partition co-efficient.

This study examined short-term exposure effects through acute toxicity assessments and comprehensive in silico analysis to identify the most promising hub gene and determine the molecular mechanisms of PGV-5 and HGV-5 for developing curcumin analogs as potential treatments for resistance mechanisms in cancer. Preliminary ADMET studies in drug development facilitate the identification of molecules with detrimental pharmacokinetic and toxicological properties before costly clinical trials, reducing the attrition rate of pharmaceutical candidates^31^. The physicochemical parameters of the compounds are crucial for evaluating oral bioavailability, solubility, permeability, and lipophilicity characteristics (Table 1; Fig. 3). These two compounds are structural analogs, differing only in their core structures: PGV-5 contains a cyclopentanone core, while HGV-5 has a cyclohexanone core. Consequently, they exhibit similar physicochemical properties, with slight but notable differences in logS (PGV-5: −4.734; HGV-5: −4.564) and logD (PGV-5: 2.943; HGV-5: 2.81) (Fig. 3). Only a single property may exceed the acceptable range, as exceeding this limit contravenes the Lipinski Rule. The MW, number of nRing, fChar, MaxRing, nRing, nRot, TPSA, nHD, nHA, logD, and logP within defined parameters suggest satisfactory oral bioavailability, promoting absorption, distribution, metabolism, and excretion to the site of action for pharmacological efficacy. However, logS characteristic surpassing the lower threshold signifies low solubility, hindering effective plasma concentrations. Formulation modification, such as solid dispersion techniques, salt formation, co-crystallization, surfactant utilization, lipid-based formulations, and complexation can be used to address this issue^32–34^.

**Table 1:** Physicochemical properties of PGV-5 and HGV-5.

Physicochemical property	Threshold	Compounds	
		PGV-5	HGV-5
Molecular weight	Optimal: 100–600	412.15	426.17
Volume	Van der Waals volume	418.498	435.79
nHA	Optimal: 0–12	7.0	7.0
nHD	Optimal: 0–7	2.0	2.0
nRot	Optimal 0–11	6.0	6.0
TPSA (Å)	Optimal 0-140	94.45	94.45
logS	The logarithm of aqueous solubility value	− 4.734	− 4.564
logP	The logarithm of the n-octanol/water distribution coefficient at pH = 7.4	2.953	2.862

Lipinski’s Rule of Five suggests that a compound’s is more likely to be inadequate if it exceeds specific criteria, including a MW of less than 500 daltons, nHD below 5, nHa not exceeding 10, molar refractivity between 40 and 130, and a logP that does not surpass 5^35^. PGV-5 and HGV-5 satisfy these criteria, exhibiting variations not exceeding 1, rendering them absorbable and capable of undergoing phase 1 metabolism in hepatic circulation. As shown in Table 2, both compounds scored 1 under Lipinski’s Rule confirming their adherence to all thresholds. According to Pfizer guidelines, compounds with logP > 3 and a topological surface area (TPSA) < 75 are more prone to toxicity^36^. A score of 1 means both PGV-5 and HGV-5 satisfies both thresholds. Similarly, GSK’s rule posits that molecules with a MW < 400 Da and logP < 4 are more likely to exhibit a favorable ADMET profile^37^. Both compounds meet these requirements, reflected by their score of 1. The Golden Triangle Rule, which assesses metabolic stability, permeability, and potency consider MW (200–500 Da) and logD between − 2 and 5 as ideal parameters^38^. A score of 0 suggest that both PGV-5 and HGV-5 conform to these requirements, making them promising drug candidates. Quantitative Estimate of Drug-likeness (QED) evaluates based on their physicochemical similarity to marketed oral drugs, providing a normalized score (0: unfavorable; 1: ideal) to prioritize candidates with favorable drug-like properties including MW, logP, nHD, nHA, PSA, nRot, aromatic rings (AROM), and structural alerts^39^. Ideal value of QED for oral drug is > 0.6 and both compounds satisfy this threshold^40,41^. The Natural Product-likeness (NP) score evaluates structural resemblance to natural products, with higher NP score indicating greater similarity. PGV-5 and HGV-5 exhibit score near 0, suggesting closer similarity to synthetic drugs rather than natural products^42^. A chelating rule score of 1 indicates that both compounds have the potential act as chelators, likely due to their phenolic moieties, which may facilitate complexation with metal ions. This property could influence their biological activity, particularly in enzymes that require metal cofactors such as zinc (Zn) or iron (Fe)^43^. Overall, PGV-5 and HGV-5 demonstrate strong potential as drug candidates, as they satisfy multiple drug-likeness criteria, including Lipinski, Pfizer, GSK, and Golden Triangle rules, with relatively high QED scores (> 0.6). However, their predicted chelating properties may influence biological interactions, particularly with metalloenzymes.

**Table 2:** Drug-likeness properties of PGV-5 and HGV-5.

Medicinal chemistry	Threshold	Compounds	
		PGV-5	HGV-5
QED	Attractive: < 0.67 Unattractive: 0.49–0.67 Complex: < 0.34	0.694	0.663
NPscore	Typically range from − 5 to 5	0.137	0.101
Lipinski rule	$\:\text{M}\text{W}\le\:500;\:\text{l}\text{o}\text{g}\text{P}\le\:5;\:\text{H}\text{a}\text{c}\text{c}\le\:10;\:\text{H}\text{d}\text{o}\text{n}\le\:$5	0.0	0.0
Pfizer rule	logP > 3; TPSA < 75	0.0	0.0
GSK rule	$\:\text{M}\text{W}\le\:400;\:\text{l}\text{o}\text{g}\text{P}\le\:$4	1.0	1.0
Golden triangle	200$\:\le\:$MW$\:\le\:$50; -2$\:\le\:$logD$\:\le\:$5	0.0	0.0
Chelator rule	–	1	1

Table 3 delineates the bioavailability of PGV-5 and HGV-5, alongside absorption parameters including Caco-2 and MDCK permeability, P-glycoprotein inhibitor and substrate capacity, human intestinal absorption (HIA), and F20% and F30% bioavailability. The Caco-2 human colon epithelial cancer cell line serves as a model for drug absorption, and all compounds exhibited good Caco-2 permeability. The transfection of the MDR1 gene into Madin-Darby Canine Kidney (MDCK) cells produces MDCK-MDR1 cells that express the efflux protein P-gp which can be used to determine if a chemical is transported through active efflux. MDCK-MDR1 cells serve as a dependable indicator of blood-brain barrier permeability, augmenting intestinal permeability as a predictor of blood-brain barrier permeability^44^. PGV-5 and HGV-5 exhibited low MDCK permeability and high P-gp inhibitory activity which could help overcome resistance mechanisms and enhance therapeutic outcomes due to increased intracellular concentrations that can enhance the cytotoxic effects of anticancer treatments^45^. In silico studies including molecular docking and network pharmacology may be performed as a preliminary screening phase before more extensive research, including in vitro and in vivo experiments.

**Table 3:** Absorption profile of PGV-5 and HGV-5.

Absorption	Threshold	Compounds	
		PGV-5	HGV-5
Caco-2 permeability	Optimal: >-5.15 log unit	− 5.259	− 5.078
MDCK permeability	Low permeability: <2 × 10 − ^6^ Medium permeability: 2–20 × 10^−6^ High passive permeability: >20 × 10^−6^	− 4.94	− 4.96
P-gp inhibitor	Category 1: inhibitor Category 0: non-inhibitor	0.992	0.947
P-gp substrate	Category 1: susbtrate Category 0: non-susbtrate	0.0	0.004
HIA	Category 1: HIA+ (HIA < 30%); Category 0: HIA- (HIA $\:\ge\:$ 30% The output value is the probability of being HIA+	0.0	0.0
F20%	Category 1: F20%+ (bioavailability < 20%) Category 2: F20%- (bioavailability $\:\ge\:$ 20%) The output value is the probability of being F20%+	0.003	0.062
F30%	Category 1: F30%+ (bioavailability < 30%) Category 2: F30%- (bioavailability $\:\ge\:$ 30%) The output value is the probability of being F30%+	0.001	0.216

Table 4 presents the distribution and metabolic characteristics of PGV-5 and HGV-5. Both compounds exhibit Plasma Protein Binding (PPB) exceeding 90%, indicating a high affinity for plasma proteins. A compound with such a high PPB may have a low therapeutic index, potentially limiting its clinical efficacy. Several strategies can be employed to address this challenge, including formulation approaches and drug delivery systems such as nanoparticles or liposomes, which encapsulate the drug to minimize direct interaction with plasma proteins and enhance bioavailability^46^. Additionally, Liu et al. (2011) emphasized that drug discovery efforts should prioritize reducing clearance, as high PPB does not necessarily restrict the unbound plasma concentration in vivo; rather, hepatic intrinsic clearance plays a more significant role. Optimizing clearance properties and minimizing efflux transporters at blood-tissue barriers can further improve unbound drug availability^47^. The compound’s volume distribution was below permitted limits (optimal: 0.04–20 L/kg), indicating that a significant portion of the drugs remains in the plasma rather than being widely distributed in body tissues, leading it to high plasma concentrations, which can increase the risk of dose dependent-toxicity. Techniques like incorporation of nanoparticles, micelles, or liposomes can be utilized to help encapsulate drugs, facilitating their release at target sites and improving bioavailability while minimizing systemic exposure. Also, developing targeted delivery systems that exploit specific receptors or transporters can enhance the accumulation of drugs in desired tissue while reducing overall systemic exposure^48^. The Blood-Brain Barrier (BBB) is a specialized structure that enables essential nutrients to flow while obstructing harmful substances into the brain. The blood-brain barrier’s restricted permeability makes it a significant obstacle in administering therapeutic drugs to the central nervous system^49^. All drugs exhibited minimal blood-brain barrier penetration. The pharmacological action of PGV-5 and HGV-5 is affected by the introduction of substrates or inhibition of cytochrome enzymes, which are crucial in drug metabolism. However, both compounds exhibited neither inhibition nor substrate activity towards cytochrome, except for CYP1A2, CYP2C8, CYP2C19, and CYP2C9 inhibitors. The inhibition of numerous CYP enzymes can substantially affect drug metabolism and pharmacokinetics, resulting in altered plasma concentrations and potential toxicity.

**Table 4:** Distribution and metabolism profile of PGV-5 and HGV-5.

Distribution	Threshold	Compounds	
		PGV-5	HGV-5
PPB	Optimal: <90%	95.745	96.86
VD	Optimal: 0.04–20 L/kg	− 0.479	− 0.458
BBB penetration	Category 1: BBB+ Category 0: BBB- The output value is the probability of being BBB+	0.003	0.003
Fu (%)	Low: <5% Middle: 5–20% High: $\:>$20%	4.126	2.876

Metabolism	Comment	Compounds	
		PGV-5	HGV-5
CYP1A2 inhibitor	Category 1: Inhibitor Category 0: Non-inhibitor	0.939	1.0
CYP1A2 substrate	Category 1: Substrate Category 0: Non-substrate	0.041	0.016
CYP2C19 inhibitor	Category 1: Inhibitor Category 0: Non-inhibitor	0.069	0.933
CYP2C19 substrate	Category 1: Substrate Category 0: Non-substrate	0.642	0.017
CYP2C9 inhibitor	Category 1: Inhibitor Category 0: Non-inhibitor	0.391	0.857
CYP2C9 substrate	Category 1: Substrate Category 0: Non-substrate	0.002	0.001
CYP2D6 inhibitor	Category 1: Inhibitor Category 0: Non-inhibitor	0.0	0.007
CYP2D6 substrate	Category 1: Substrate Category 0: Non-substrate	0.0	0.027
CYP3A4 inhibitor	Category 1: Inhibitor Category 0: Non-inhibitor	0.026	0.0
CYP3A4 substrate	Category 1: Substrate Category 0: Non-substrate	0.0	0.0

Table 5 presents the excretion and toxicity profiles of PGV-5 and HGV-5, demonstrating moderate clearance and an ultra-short half-life. This rapid elimination may reduce therapeutic efficacy and necessitate frequent dosing. Various strategies can be employed to address this limitation, particularly through formulation innovations such as sustained-release systems utilizing polymer-based matrices or nanoparticulate carriers. These approaches help regulate the drug release rate while maintaining a stable plasma concentration^50^. Despite the short half-life, the clearance rate remains within an acceptable range. Additionally, both compounds exhibited a favorable toxicity profile, with no significant drug-induced liver damage, AMES mutagenicity, acute oral toxicity in rats, skin sensitization, ocular corrosion, ocular irritation, respiratory toxicity, or genotoxicity.

**Table 5:** Excretion and toxicity profile of PGV-5 and HGV-5.

Excretion	Threshold	Compounds	
		PGV-5	HGV-5
CL	High clearance: >15 ml/min/kg Moderate clearance: 5–15 ml/min/kg Low clearance: <5 ml/min/kg	7.328	7.067
T1/2	Ultra-short half-life: 0.5–1 h Short half-life: 1–4 h Intermediate short half-life: 4–8 h Long half-life: >8 h	0.821	0.783

Toxicity	Threshold	Compounds	
		PGV-5	HGV-5
DILI	Category 1: High risk of DILI Category 0: No risk of DILI	0.425	0.304
AMES toxicity	Category 1: Ames positive Category 0: Ames negative	0.309	0.255
Rat oral acute toxicity	Category 1: Low-toxicity Category 0: High-toxicity	0.431	0.266
FDAMDD	Category 1: FDAMDD + Category 0: FDAMDD – The output value is the probability of being positive	0.688	0.793
Skin sensitization	Category 1: Sensitizer Category 0: Non-sensitizer	0.436	0.464
Eye corrosion	Category 1: Corrosive Category 0: Non-corrosive	0.001	0.001
Eye irritation	Category 1: Irritants Category 0: Non-irritants	0.566	0.430
Respiratory toxicity	Category 1: Respiratory toxicants Category 0: Non-respiratory toxicants	0.557	0.507

### Acute-toxicity studies

This study aimed to evaluate the short-term toxicity of PGV-5 and HGV-5. The findings provide crucial data on the immediate toxic effects of these compounds, aiding in the identification of potential risks in human use. Additionally, the study helps determine the maximum non-lethal dose and minimum lethal dose, which are essential for establishing a safe starting dose in first-in-human (FIH) studies^51^. The initial dosage was 300 mg/kg body weight, and the dosage was increased to 2000 mg/kg body weight resulting one mice death at PGV-5 group while all HGV-5 group survived. Consequently, in the primary test, HGV-5 is administered at a dose of 2000 mg/kg body weight, whereas PGV-5 is administered at a dosage of 300 mg/kg body weight. All mice in the HGV-5 group survived until the test’s conclusion, while one mouse died in the PGV-5 group.

Behavioral patterns of both treated animals and the control group were monitored over short intervals, followed by extended observations every 24 h. No drug-related alterations were noted in behavior, respiration, dermal effects, pelage, ocular condition, nasal appearance, urine or fecal excretion, as well as temperature. Body weight varied in both the test and control groups from days 1 to 14, with no significant weight changes related to the treatment (Table 6, Supplementary Table 1). The fluctuation in the mice weight can be explained because of body fat accumulation or normal psychological adaptation response to the compounds which lead to low appetite and food intake.

**Table 6:** Acute toxicity test result in treatment group and control group. The values represent mean ± standard error mean (SEM) from *n* = 5, no significant change compared with respective control.

Group	Days				
	0	4	8	12	14
	Body weight (g)				
Control (CMC-Na 0.7%)	33.62$\:\pm\:$0.44	30.54$\:\pm\:$1.22	34.45$\:\pm\:$0.58	34.68$\:\pm\:$0.74	34.48$\:\pm\:$0.64
Treatment (PGV-5 300 mg/kgBW)	30.38$\:\pm\:$1.04	29.75$\:\pm\:$1.59	30.91$\:\pm\:$1.47	31.54$\:\pm\:$0.96	31.27$\:\pm\:$1.01
Control (CMC-Na 0.7%)	30.12$\:\pm\:$0.27	32.94$\:\pm\:$0.92	33.24$\:\pm\:$1.08	33.16$\:\pm\:$0.99	33.24$\:\pm\:$0.84
Treatment (HGV-5 2000 mg/kgBW)	29.24$\:\pm\:$0.37	30.24$\:\pm\:$0.79	31.30$\:\pm\:$0.85	31.95$\:\pm\:$0.88	32.13$\:\pm\:$0.99

Group	Liver	Spleen	Lung	Kidney	Heart
Terminal organ weight (g)					
Control (CMC-Na 0.7%)	1.60$\:\pm\:$0.14	0.17$\:\pm\:$0.03	0.20$\:\pm\:$0.22	0.18$\:\pm\:$0.01	0.17$\:\pm\:$0.01
Treatment (PGV-5 300 mg/kgBW)	1.79$\:\pm\:$0.07	0.20$\:\pm\:$0.03	0.15$\:\pm\:$0.02	0.17$\:\pm\:$0.01	0.15$\:\pm\:$0.02
Control (CMC-Na 0.7%)	1.63$\:\pm\:$0.11	0.14$\:\pm\:$0.03	0.20$\:\pm\:$0.02	0.17$\:\pm\:$0.01	0.15$\:\pm\:$0.01
Treatment (HGV-5 2000 mg/kgBW)	1.75$\:\pm\:$0.15	0.13$\:\pm\:$0.01	0.21$\:\pm\:$0.02	0.18$\:\pm\:$0.01	0.15$\:\pm\:0$0.02
Percentage relative organ weight (%)					
Control (CMC-Na 0.7%)	4.64$\:\pm\:$0.40	0.51$\:\pm\:$0.09	0.59$\:\pm\:$0.06	0.51$\:\pm\:$0.02	0.44$\:\pm\:$0.03
Treatment (PGV-5 300 mg/kgBW)	5.75$\:\pm\:$0.38	0.64$\:\pm\:$0.11	0.49$\:\pm\:$0.06	0.54$\:\pm\:$0.05	0.47$\:\pm\:$0.05
Control (CMC-Na 0.7%)	4.87$\:\pm\:$0.21	0.41$\:\pm\:$0.07	0.60$\:\pm\:$0.06	0.52$\:\pm\:$0.03	0.44$\:\pm\:$0.04
Treatment (HGV-5 2000 mg/kgBW)	5.44$\:\pm\:$0.44	0.39$\:\pm\:$0.03	0.67$\:\pm\:$0.08	0.56$\:\pm\:$0.03	0.48$\:\pm\:$0.44

**Fig. 4:**
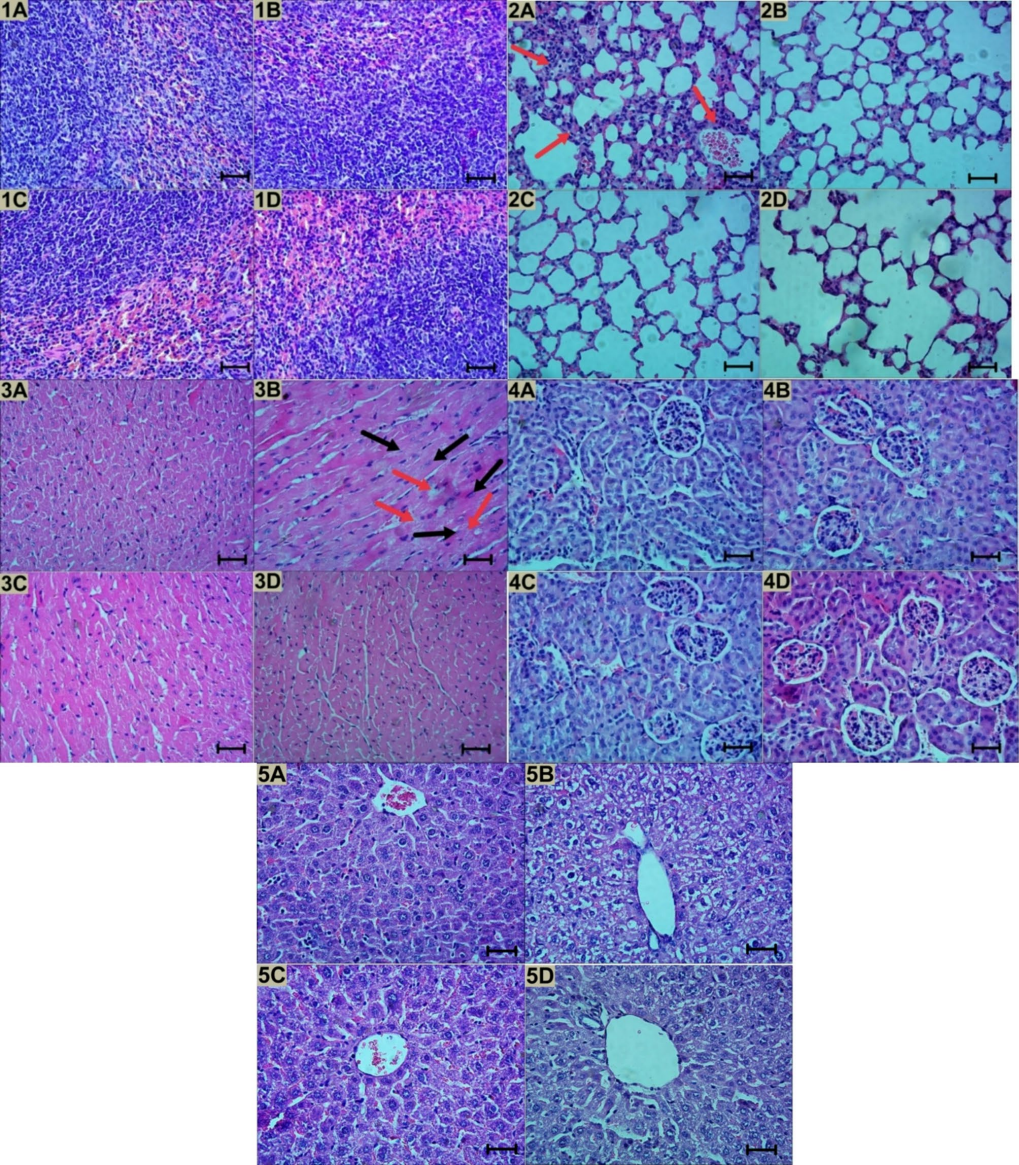
Histopathological examination in several organs, including (**1**) Spleen, (**2**) Lung, (**3**) Heart, (**4**) Kidney, and (**5**) Liver. These organs were procured following cervical dislocation. Upon fixation, processing and dehydration, the cross section of organs were stained with H&E. Representative images of organs are displayed at magnification of 400x; scale bars: 50 μm. (**A**) Treatment group receiving PGV-5 300 mg/kgBW, exhibiting normal cellular architecture except lung, indicating an interstitial alveolar edema (as indicated by red arrow); (**B**) Treatment group receiving HGV-5 2000 mg/kgBW, exhibiting normal cellular architecture except heart, indicating an hydropic degeneration (as indicated by red arrow) and karyorrhexis (as indicated by black arrow); (**C**) PGV-5 control group receiving CMC-Na 0.7%, showing normal cellular architecture of organ; (**D**) HGV-5 control group receiving CMC-Na 0.7%, showing normal cellular architecture of organ.

**Fig. 5:**
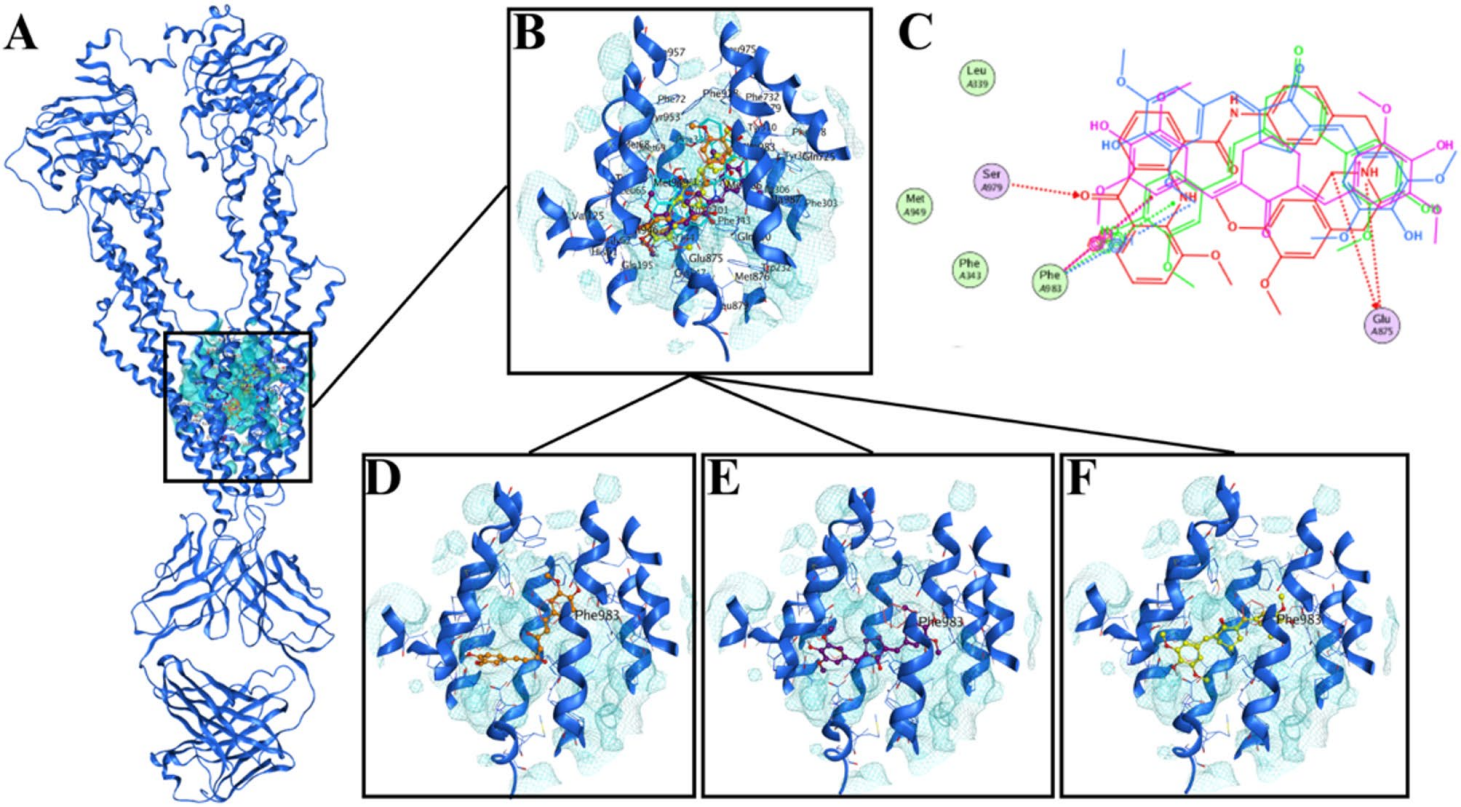
Visualization of molecular docking for PGV-5, HGV-5, and curcumin in P-gp receptor (**A**) Full ribbon view (**B**) Site view (**C**) 2D ligand interaction view (**D**) 3D curcumin interaction view (**E**) 3D PGV-5 interaction view (**F**) 3D HGV-5 interaction view. The site active or binding site are marked by the crystallographic pocket from the PDB structure.

The experimental group’s essential organs were examined for alterations compared to the control group. The HGV-5 test group showed an irregular red hue in the hearts, while the rest organs were normal. Terminal organ weights (Table 6, Supplementary Table 2) and percentages of relative organ weight (Table 6, Supplementary Table 3) were also analyzed. Microscopically, karyokinesis and hydropic degeneration were observed in the hearts of the HGV-5 test compound compared to the control group, and interstitial alveolar edema was observed in the PGV-5 test group (Figs. 4 and 5). There is no evidence that neutrophilic infiltration or cytokine release contribute to organs pathophysiological changes. According to these results, PGV-5 is placed in GHS class 4 (300 < LD_50_ < 2000 mg/kgBW) meanwhile HGV-5 is classed under GHS class 5 (2000 < LD_50_ < 5000 mg/kg BW). The observed toxicity profiles of PGV-5 and HGV-5 may be attributed to their pharmacokinetic properties, including strong P-gp inhibition, low P-gp substrate activity, high plasma protein binding (PPB), and low volume of distribution (VD). These factors can lead to increased drug accumulation in certain organs, resulting in toxicity, as evidenced by interstitial alveolar edema in the lungs and cardiac toxicity, characterized by karyokinesis and hydropic degeneration in the heart. However, with appropriate development strategies, these compounds hold potential for therapeutic application. Several approaches can be considered to mitigate toxicity and enhance efficacy, including targeted delivery systems using nanoparticles or liposomal formulations to improve specificity, combination therapy to optimize treatment effects, encapsulation techniques to minimize direct plasma protein interactions and enhance bioavailability, and sustained-release formulations to maintain therapeutic drug levels while reducing toxicity risks^52^.

### Molecular docking on P-glycoprotein

This study investigates the potential inhibitory activity of PGV-5 and HGV-5 in P-gp signaling to overcome cancer therapy resistance. The molecular docking study used the crystallographic structure of human P-gp (ABCB1) reconstituted in a nanodisc, specifically in complex with the Fab fragment of the inhibitory monoclonal antibody MRK16 and the inhibitor elacridar (R0Z). This approach was previously employed to design and construct a novel class of compounds based on the 1-phenylpyrrolol[2,1-a]isoquinoline scaffold, aimed at overcoming P-glycoprotein-mediated drug resistance by functioning as a strong inhibitor through molecular docking calculations^53^. R0Z or Elacridar was defined as the native ligand and as the binding site for docking the test.

The natural ligand R0Z exhibits affinity for specific amino acid residues in the binding area of the P-gp receptor, namely Glu875, Glu876, Ser797, and Phe98. The RMSD calculations demonstrate significant overlap, with a root-mean-square deviation of 0.28 Å. Redocking and docking were performed using MOE’s default settings: triangle matcher placement, ligand atom site, initial scoring with London dG, and final scoring employing the GBVI/WISA dG algorithm. Figure 5 presents the docking results for both compounds, encompassing their 2D and 3D representations.

The molecular docking research revealed that PGV-5 and HGV-5 exhibit binding affinities of -8.60 kJ/mol and − 8.83 kJ/mol, respectively, while curcumin demonstrated a binding affinity of -7.52 kJ/mol. These analogs show improved efficacy as P-gp inhibitors compared to curcumin. PGV-5 and HGV-5 specifically attach to the amino acid residue Phe983, which is located in a critical region of the P-gp protein involved in substrate binding and transport (Table 7). Curcumin’s ring analogues form an π-π interaction with Phe983, enhancing the blocking ability of efflux transporters and increasing intracellular concentrations of chemotherapeutic agents^45^. The hydrogen bonding and π-π resonance interactions with the Phe983 residue serve as strong predictive indicators of potent inhibition. Similar interactions have been reported in verapamil, a first-generation P-gp inhibitor, as well as in piperine and its derivatives, which exhibit comparable activity^54,55^. Additionally, interactions with Phe985 have been confirmed through molecular dynamics simulations and experimentally validated in the study by Khumbar et al. (2024), which investigated the potential of esculetin derivatives as anti-MDR agents via P-gp modulation. Targeting residues such as Phe983 with specific inhibitors not only enhances drug efficacy but also reduces the likelihood of resistance development^56^.

**Table 7:** Molecular docking result including docking score (kJ/mol) and amino acid residues interacting with PGV-5, HGV-5, and Curcumin (as a comparison) with P-gp.

Ligand	*P*-gp						
	Docking score (kJ/mol)	RMSD (Å)	Ligand atom	Receptor atom	Amino acid	Interaction	Distance
Native ligand (Elacridar)	− 10.01	0.284	C	O	Glu875	H-donor	3.07
			N	O	Glu876	H-donor	3.07
			O	O	Ser797	H-acceptor	2.89
			N	O	Glu875	Ionic	3.07
			N	O	Glu875	Ionic	3.69
			6-ring	6-ring	Phe983	Pi-pi	3.59
Curcumin	− 7.52	–	6-ring	6-ring	Phe983	Pi-pi	3.77
PGV-5	− 8.60	–	6-ring	6-ring	Phe983	Pi-pi	3.86
HGV-5	− 8.83	–	6-ring	6-ring	Phe983	Pi-pi	3.90

The result was determined by the highest docking score and the most interaction with the amino acid that overlayed between native ligand, PGV-5, HGV-5 and Curcumin.

In numerous studies, curcumin has shown promise as an anti-MDR agent in cancer by regulating P-gp function and expression. Both in vitro and in vivo studies have demonstrated that curcumin can inhibit P-gp activity, enhance the intracellular accumulation of chemotherapeutic drugs, and improve cancer cell sensitivity to treatment. These effects have been observed in various MDR cancer cell lines, including cervical, gastric, and colorectal carcinoma, as well as chronic myeloid leukemia^9,57–59^. Furthermore, curcumin downregulates P-gp expression by inhibiting the PI3K/Akt/NF-κB pathway and modulating microRNAs, thereby reducing drug resistance. Animal model studies further corroborate these findings, showing that curcumin administration leads to decreased MDR1 gene expression and lower P-gp protein levels^51^.

PGV-5 and HGV-5 exhibiting strong P-gp inhibitory activity coupled with low P-gp substrate affinity (Table 3) show significant potential in overcoming multidrug resistance in cancer. By inhibiting the ATP-dependent efflux function of P-gp without acting as its substrates, these compounds can avoid P-gp-mediated drug expulsion, thereby maintaining their intracellular concentration and therapeutic effectiveness^45,60^. This potential is further supported by molecular docking results, which demonstrate favorable binding interactions and suggest that both compounds are viable candidates for development as anticancer agents targeting multidrug resistance. To further elucidate their mechanism of action, network pharmacology analysis may be employed to identify relevant molecular targets involved in cancer multidrug resistance that are influenced by these compounds.

### Molecular Dynamics Simulation

**Fig. 6:**
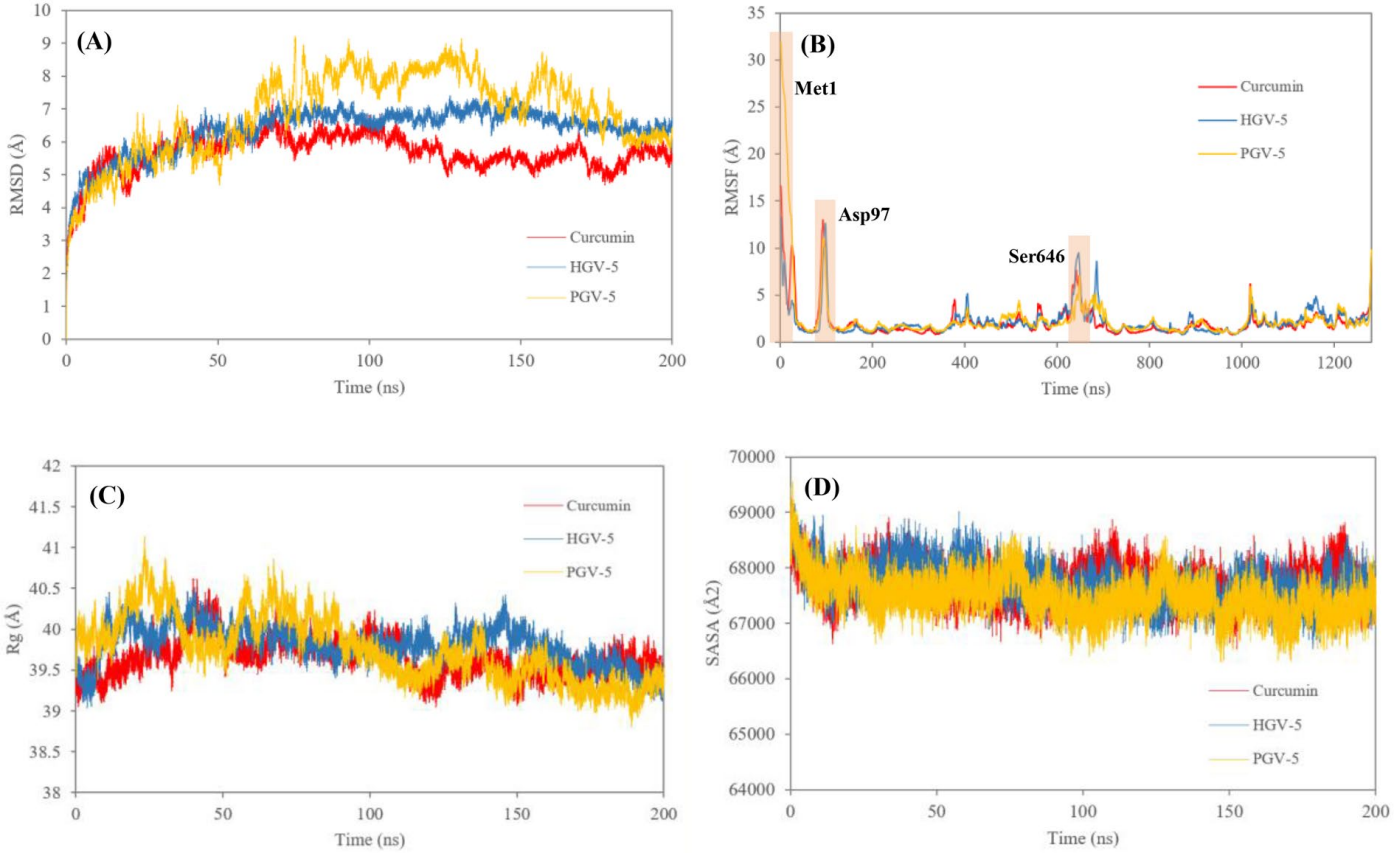
Plots of (**A**) RMSD, indicating the stability of the protein-ligand complex; (**B**) RMSF, exhibiting the flexibility of specific residues; (**C**) Rg, showing the compactness; and (**D**) SASA, illustrating the protein's surface being exposed to solvent molecules during the simulation of curcumin (red), HGV-5 (blue), and PGV-5 (yellow) with P-gp during the 200 ns of MD simulations

The stability of protein-ligand complexes obtained from molecular docking was evaluated through molecular dynamics (MD) simulations using parameters such as RMSD, RMSF, Rg, SASA, hydrogen bonds, and MM-PBSA. The comparative stability of curcumin and its analogues (HGV-5 and PGV-5) bound to P-glycoprotein (P-gp) is illustrated in Fig. 6.

RMSD (root mean square deviation) was employed to assess the structural stability of the protein backbone throughout the 200 ns simulation. Lower RMSD values indicate higher stability. All complexes exhibited elevated RMSD values (>2.00 Å) within the first 0.7 ns, reflecting early structural adjustments and less stable conformations (Fig 6A). The average RMSD values were 5.66 Å for curcumin, 6.35 Å for HGV-5, and 6.85 Å for PGV-5. Among these, curcumin-P-gp complex showed the most stable behavior with minimal fluctuations, followed by HGV-5 with moderate stability. PGV-5, however, displayed increasing RMSD, surpassing 7.0 Å after 60 ns and reaching 9.23 Å at ~75 ns, suggesting greater conformational changes and less stable binding. Overall, the results suggest that curcumin and HGV-5 form more stable interactions with P-gp compared to PGV-5, supporting their potential as better inhibitors during the 200 ns MD simulation.

RMSF analysis was conducted to evaluate the flexibility of specific residues within the protein-ligand complexes during the simulation. Higher RMSF values indicate greater residue mobility, while lower values reflect structural stability. As shown in Fig. 6B, most residues showed limited fluctuation, with notable exceptions at Met1, Asp97, and Ser646—regions distant from the binding pocket and unlikely to impact ligand binding. Conversely, key interacting residues remained stable throughout.

Rg reflects the compactness of the protein structure, providing insight into its overall size and shape. Lower Rg values indicate a more compact, globular conformation, whereas higher values suggest a more extended and flexible structure. As shown in Fig. 6C, the Rg values ranged from 39 to 40.5 Å. These relatively high values indicate flexible protein behavior, which may indicate structural instability or partial unfolding. The observed flexibility could also be attributed to the inherent size and shape of the protein, allowing it to explore a broader range of conformations during the simulation. 

SASA analysis was conducted to evaluate the degree of surface exposure of the protein-ligand complexes to the solvent, which reflects protein folding, stability, and interaction characteristics. Higher SASA values indicate expanded surface areas, while lower values suggest a more compact structure. All complexes stabilized after 30 ns and maintained consistent SASA values thereafter (Fig. 6D). Curcumin showed the highest SASA (67,727 Å²), followed closely by HGV-5 (67,719 Å²) and PGV-5 (67,502 Å²), suggesting that curcumin may form a slightly more rigid and exposed complex with P-gp., potentially indicating stronger and more stable interactions.

**Fig. 7:**
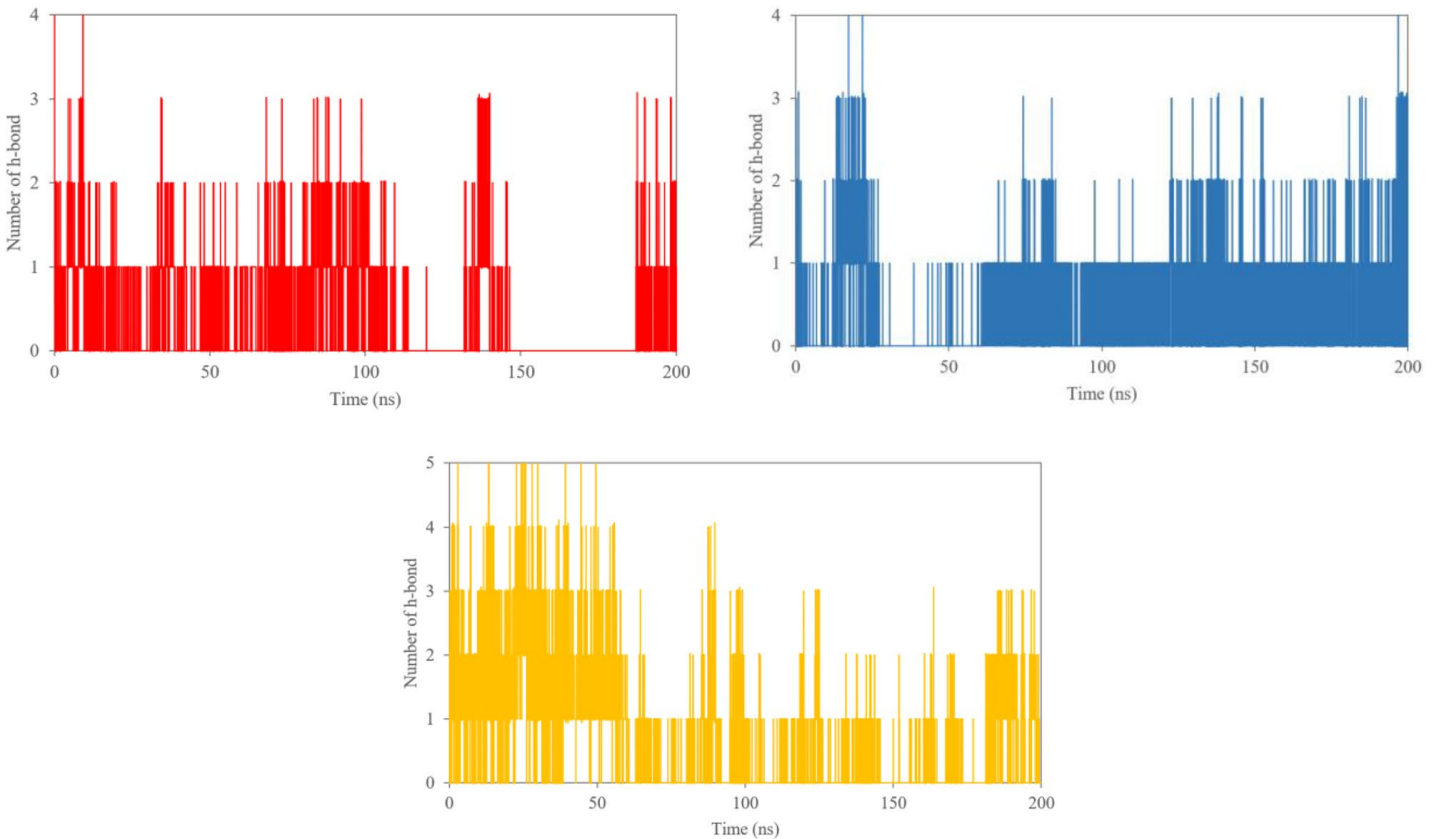
Hydrogen-bonds plot of curcumin (red), HGV-5 (blue), and PGV-5 (yellow) with P-gp during the 200 ns of MD simulations

Hydrogen bonds, as noncovalent interactions, are essential in evaluating the stability and functionality of molecular systems in MD simulations. Figure 7 shows the number of hydrogen bonds formed throughout the simulation. PGV-5 initially formed the highest number, reaching five, while curcumin and HGV-5 maintained a maximum of four. However, the presence of more hydrogen bonds at the start did not directly correlate with greater overall stability, as seen in PGV-5's fluctuating RMSD.

**Fig. 8:**
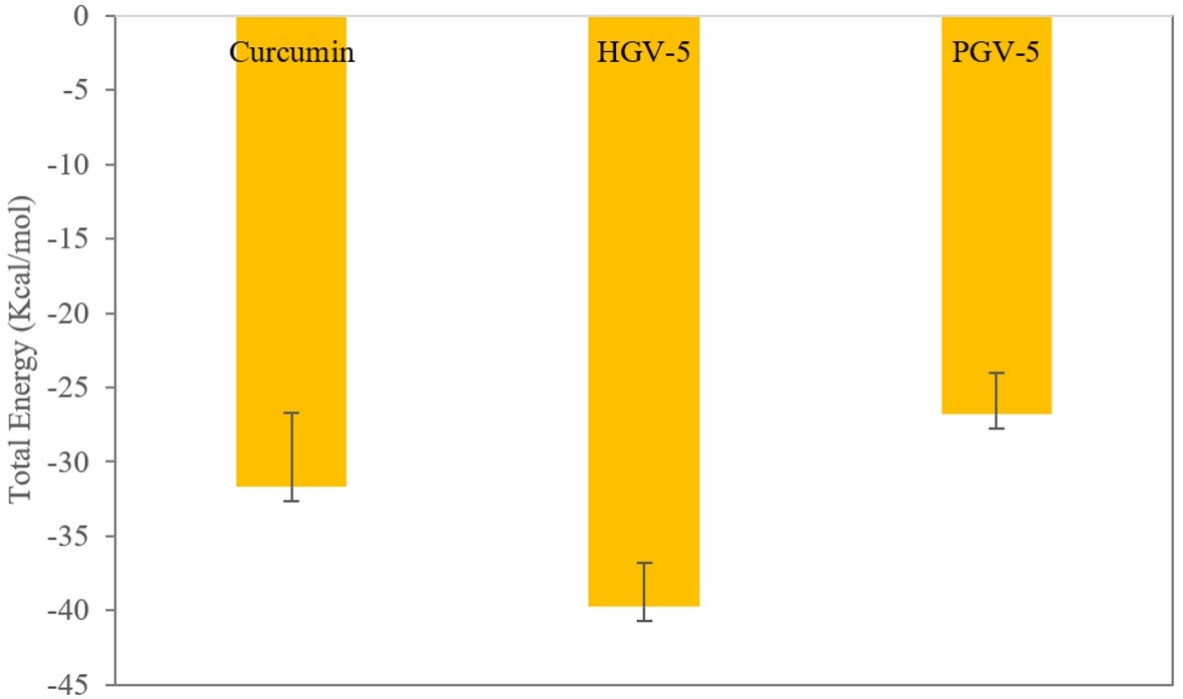
Plots of the total energy of protein-ligand complexes during the MD simulations

**Fig. 9:**
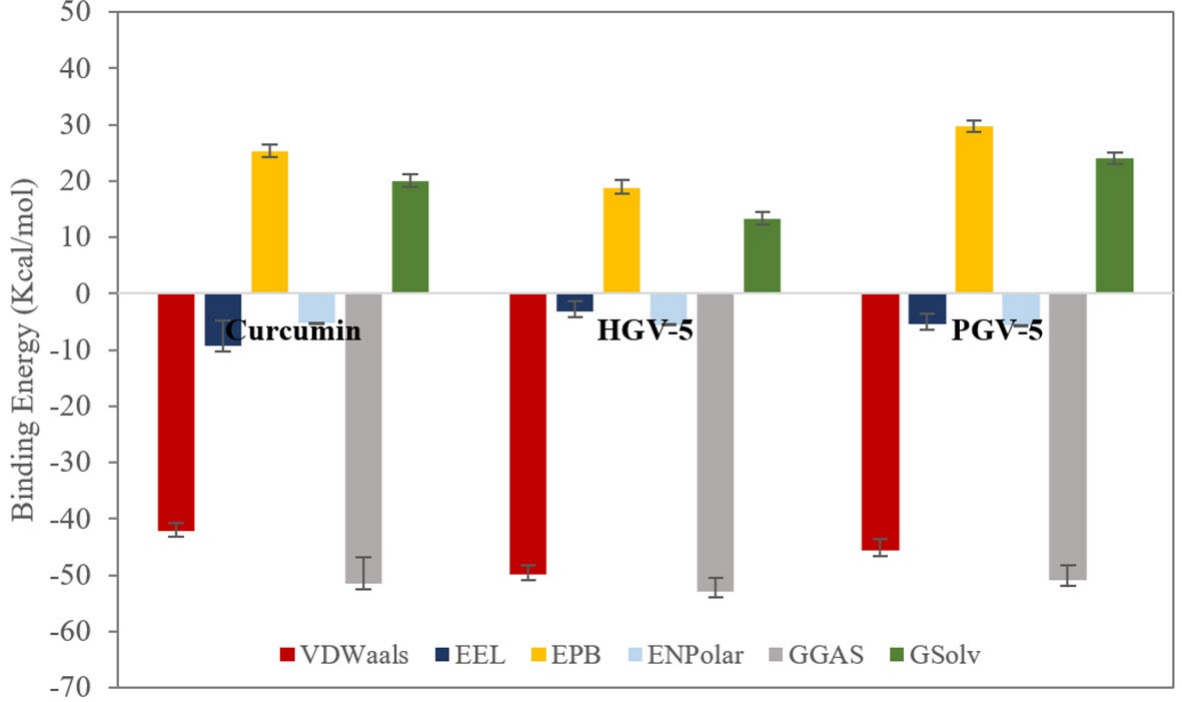
Plots of binding energy calculated based on the MM-PBSA analysis concerning the interaction of the ligands with the P-gp protein.

To further quantify binding affinity, MM-PBSA analysis was performed on snapshots between 75–150 ns, when systems reached equilibrium based on RMSD trends (Figs. 8 and 9). This approach provides robust estimates of binding free energy and its components. Recognized for its balance of accuracy and efficiency, MM-PBSA is widely used in protein-ligand interaction studies within pharmaceutical research. HGV-5 exhibited the most favorable total binding free energy (ΔGtotal) at −39.71 ± 2.89 kcal/mol, indicating its strong potential as a protein inhibitor, followed by curcumin (−31.64 ± 4.97 kcal/mol) and PGV-5 (−26.80 ± 2.78 kcal/mol).

The average binding free energies were further decomposed into individual energy components, including Van der Waals (ΔVdW), average electrostatic (ΔEEL), Poisson-Boltzmann energy (ΔEPB) polar energy (ΔENPolar), gas-energy(ΔGGas), and solvation (ΔGSolv) energies (Supplementary Table 9). These energetic contributions collectively stabilized the protein-ligand complexes, as illustrated in Fig. 9. As illustrated in Fig. 9, vdW, ΔELL, ΔENPolar, and ΔGGas components contribute favorably to the stability of the protein-ligand complex by lowering the binding free energy. In contrast, ΔEPB and ΔGSolv exhibit destabilizing effects by increasing the overall free energy. Notably, the binding free energies from MD were more negative than those from molecular docking, confirming the persistence and favorable interaction of all three ligands—particularly HGV-5 and curcumin—with P-gp throughout the simulation.

### Network pharmacology

**Fig. 10:**
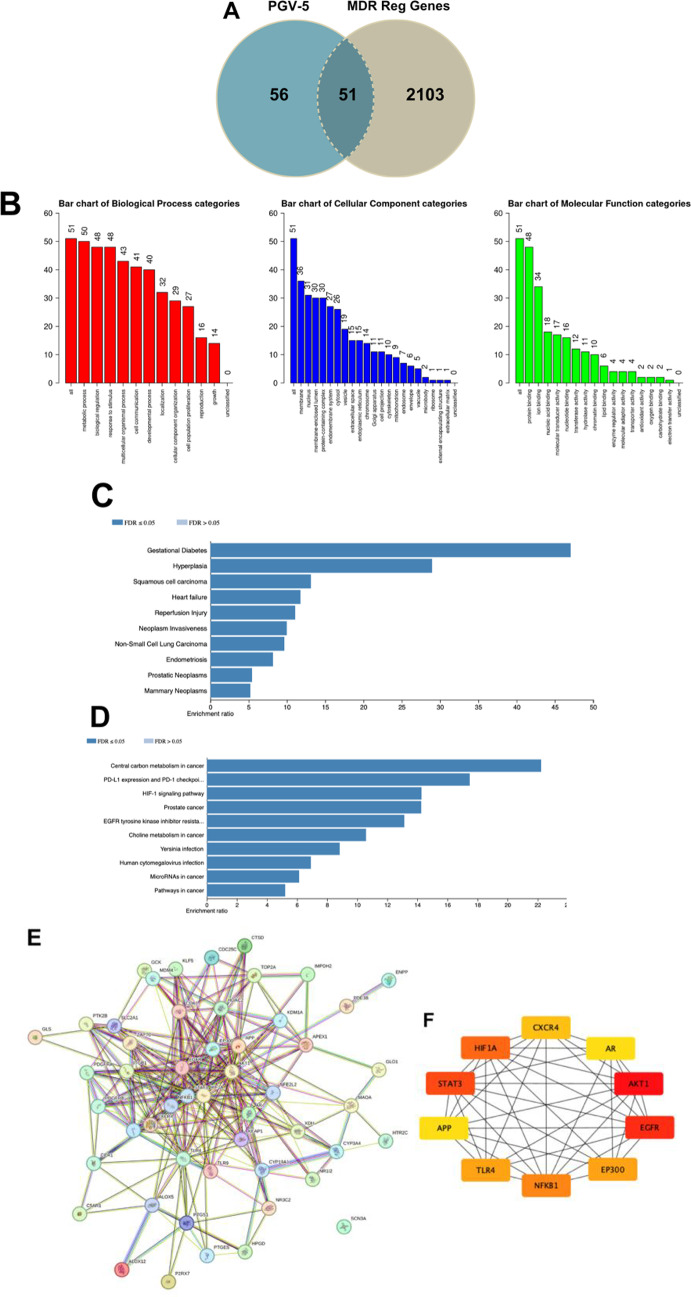
(**A**) Venn diagram illustrating 51 potential target gene identified as PTPMs. (**B**) Functional annotation enrichment analysis of PTPMs classified into Biological Process (BP), Cellular Component (CC), and Molecular Function (MF). (**C**) Analysis of disease-gene association for PTPMs. (**D**) KEGG pathway enrichment analysis, light blue indicates signifies FDR > 0.05 while dark blue indicates FDR < 0.05. (**E**) Protein-protein interaction of PTPMs as derived from STRING comprises of 51 nodes with 258 edges. The average local clustering coefficient is 0.61, indicating a significant degree of connectivity among PTPMs. (**F**) Top 10 potential target gene based on degree score, as evaluated by CytoHubba plugin of Cytoscape. The color of each node corresponds to its degree score, with red signifying the highest score, followed by orange and yellow.

The bioinformatics study used network pharmacology to understand the relationships between diseases, genes, drugs, and KEGG pathways. It identified new treatment targets, pathways, and putative hub genes affected by PGV-5 and HGV-5 in key processes associated with multidrug resistance in cancer, which might be employed for future research^61^. The Swiss Target Prediction page indicates that target fishing for PGV-5 identifies 19 targets whereas HGV-5 shows 21 receptor targets (Supplementary Table 4). The SuperPred Target Prediction page indicates 94 receptor targets for PGV-5 and 96 receptor targets for HGV-5 (Supplementary Tables 5, 6). GeneCards provided 2154 regulatory genes associated with multidrug resistance in cancer (Supplementary Table 7). The intersecting gene visualized based on Venn diagram collected 51 potential target genes, known as PTPMs for PGV-5 whereas HGV-5 collected 53 potential target genes, known as PTHMs. Both potential target genes were included in further analysis (Figs. 10A and 10A).

**Fig. 11:**
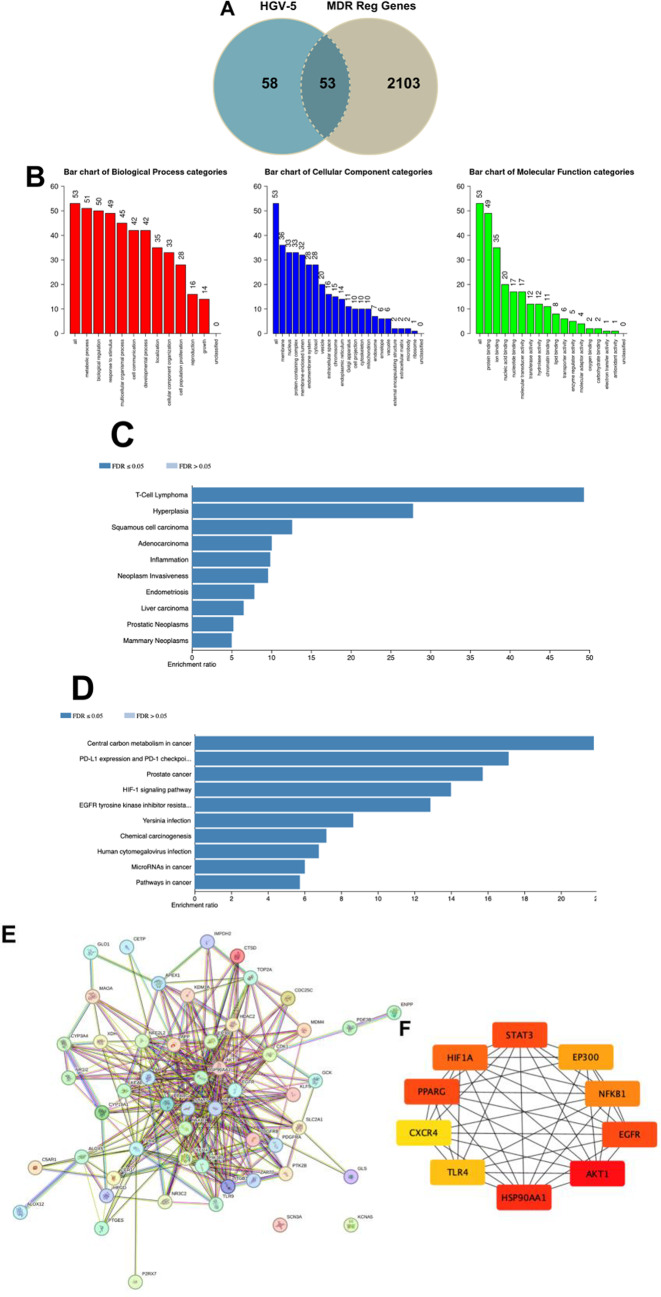
(**A**) Venn diagram illustrating 53 potential target gene identified as PTHMs. (B) Functional annotation enrichment analysis of PTHMs classified into Biological Process (BP), Cellular Component (CC), and Molecular Function (MF). (**C**) Analysis of disease-gene association for PTHMs. (**D**) KEGG pathway enrichment analysis, light blue indicates signifies FDR > 0.05 while dark blue indicates FDR < 0.05. (**E**) Protein-protein interaction of PTHMs as derived from STRING comprises of 53 nodes with 307 edges. The average local clustering coefficient is 0.64, indicating a significant degree of connectivity among PTHMs. (**F**) Top 10 potential target gene based on degree score, as evaluated by CytoHubba plugin of Cytoscape. The color of each node corresponds to its degree score, with red signifying the highest score, followed by orange and yellow.

Gene ontology analyses were performed to enrich the annotation of PTPMs and PTHMs, categorized into into Biological Process (BP), Cellular Component (CC), and Molecular Function (MF) (Figs. 10B and 10B). PGV-5 and HGV-5 revealed several genes involved in these processes. Disease enrichment analyses based on DisGenet database revealed a relationship between PTPMs and gestational diabetes and hyperplasia, whereas PTHMs investigation identified T-cell lymphoma and hyperplasia as the leading two based on enrichment ratio. Gestational Diabetes Mellitus (GDM) is linked to an increased risk of several malignancies of cancer. Insulin resistance results in prolonged hyperinsulinemia, which may enhance cellular proliferation and suppress apoptosis, thus facilitating cancer. Additionally, elevated inflammatory markers may contribute to cancer progression^62,63^ (Figs. 10C and 10C). KEGG pathways revealed that both PTPMs and PTHMs involved in several pathways including central carbon metabolism in cancer and PDL-1 expression and PD-1 checkpoint. These factors regulate metabolic reprogramming in cancer cells, enabling them to adapt to diverse environmental situations. The interaction between PD-1 and PD-L1 is essential for the immune evasion of cancer cells, involving interferon-gamma, PI3K/AKT, and MAPK signaling pathways. The interaction of PD-L1 with tumor cells obstructs T-cell receptor signaling, hence decreasing T-cell activation and proliferation, and impairing their capacity to identify and eradicate cancer cells^64,65^. (Figs. 10D and 11D).

**Fig. 12:**
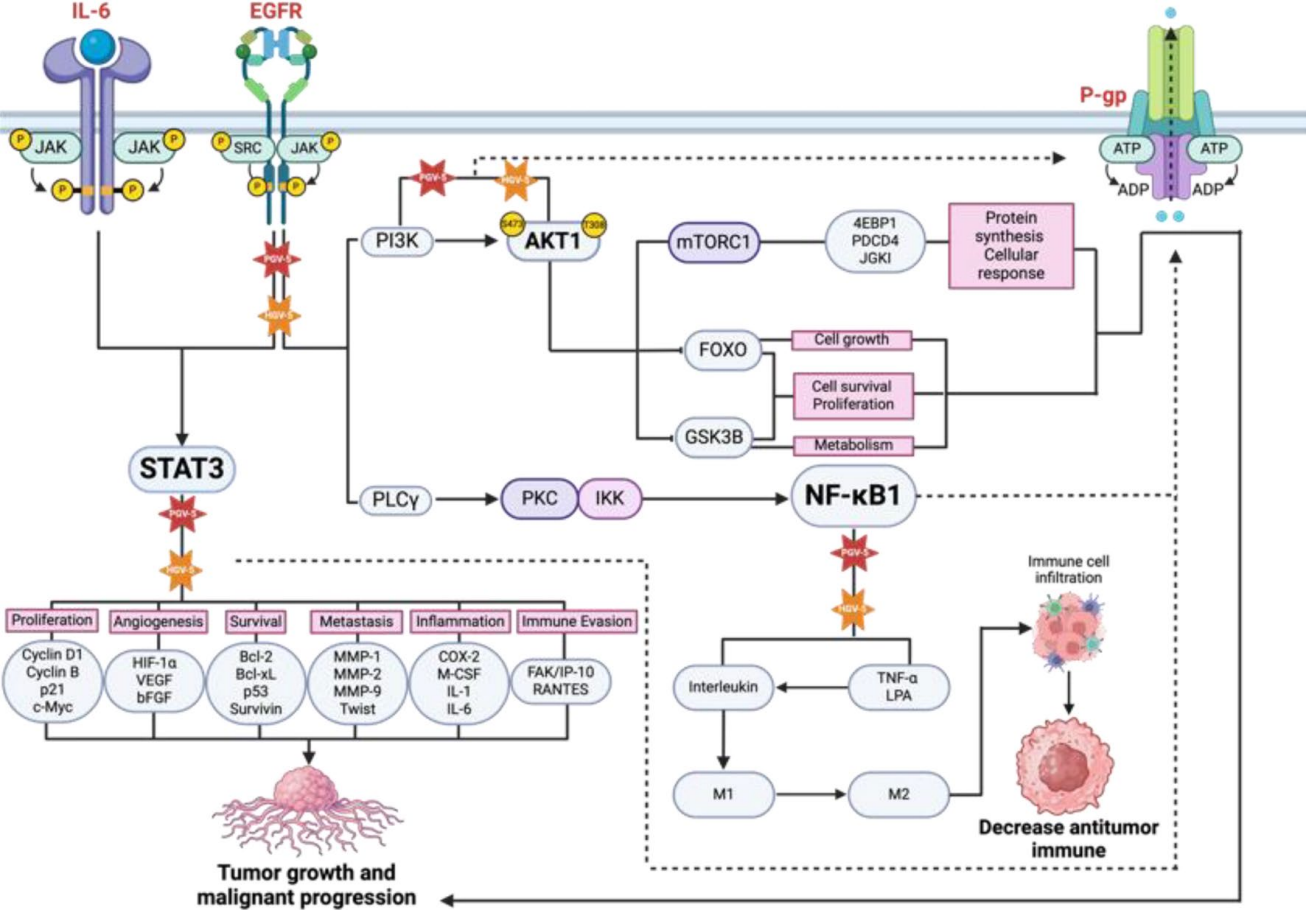
The proposed mechanism of PGV-5 and HGV-5 blocking for major potential target (STAT3, AKT1, EGFR, and NF$\:\kappa\:$B1) in overcoming multidrug resistance in cancer. Dotted arrow indicated interplay signaling.

PPI network illustrating the interconnections among the proteins (Figs. 10E and 11E, Supplementary Table 8). The hub gene of PGV-5 was identified, with the top 10 genes based on degree score: AKT1, EGFR, STAT3, HIF1A, NF-κB1, TLR4, EP300, CXCR4, APP, and AR. The top genes for HGV-5 were AKT1, HSP90AA1, STAT3, EGFR, PPARG, HIF1A, NF-κB1, EP300, TLR4, and CXCR4 (Table 8; Figs. 10F and 11F). Bioinformatic analysis shows that PGV-5 and HGV-5 holds significant potential in overcoming multidrug resistance in cancer through 4 principal proteins identified on high degree score: AKT1, STAT3, EGFR, and NF-κB1. These proteins play a significant role in signaling pathways, cell survival, growth, and drug resistance gene regulation (Fig. 12).

**Table 8:** Top ten potential gene targets for PGV-5 and HGV-5 as anti-multidrug resistance in cancer identified by PPI networks, ranked by degree score which reflect the amounts of connections each protein has within the network, with a higher score signifying greater connectivity among each gene.

Rank	Gene symbol	Gene name	Degree score
*PGV-5*			
1	AKT1	AKT Serine/Threonine Kinase 1	37
2	EGFR	Epidermal Growth Factor Receptor	29
3	STAT3	Signal Transducer and Activator of Transcription 3	28
4	HIF1A	Hypoxia Inducible Factor 1 Subunit Alpha	24
5	NF-κB1	Nuclear Factor Kappa B Subunit 1	23
6	TLR4	Toll-Like Receptor 4	21
6	EP300	E1A Binding Protein P300	21
8	CXCR4	C-X-C Motif Chemokine Receptor 4	17
9	APP	Amyloid Beta Precursor Protein	16
9	AR	Androgen Receptor	16
*HGV-5*			
1	AKT1	AKT Serine/Threonine Kinase 1	38
2	HSP90AA1	Heat Shock Protein 90 Alpha Family Class A Member 1	31
3	STAT3	Signal Transducer and Activator of Transcription 3	30
3	EGFR	Epidermal Growth Factor Receptor	30
3	PPARG	Peroxisome Proliferator Activated Receptor Gamma	30
6	HIF1A	Hypoxia Inducible factor 1 Subunit Alpha	26
7	NF-κB1	Nuclear Factor Kappa B Subunit 1	24
8	EP300	E1A Binding Protein P300	23
9	TLR4	Toll-Like Receptor 4	22
10	CXCR4	C-X-C Motif Chemokine Receptor 4	19

Epidermal Growth Factor Receptor (EGFR) signaling pathway involves dimerization and autophosphorylation, triggering downstream signaling cascades that promotes cell proliferation, survival, and migration. These pathways, including RAS-RAF-MAPK Pathway, PI3K-AKT Pathway, and JAK-STAT Pathway regulate gene expression related to cell proliferation and differentiation, playing a crucial role in tumor invasion and metastasis^66^. AKT promotes cell survival by phosphorylating transcription factors that inhibit apoptosis such as BAD and FOXO and activating mTOR, for protein synthesis and cell growth^66,67^. The JAK/STAT pathway, activated by EGFR signaling, influences transcription factor activity, affecting genes related to cell survival and proliferation, contributing to oncogenic process in various cancers^68,69^. Inhibiting this pathway can lead to death of cancer cell.

AKT1 is a crucial component of the PI3K/AKT signaling pathway, activated by growth factors. It is activated through phosphorylation at Thr308 (T308) and Ser473 (S473) which activates mTORC1 complex, affecting transcription factors like 4E-BP1, PDCD4, and JGKI. Dysregulation of these pathways can lead to tumor growth, resistance to therapies, and enhanced survival of cancer cells. AKT1 also inhibits apoptosis and promotes cellular growth through phosphorylation of tumor suppressor gene FOXO and GSK3$\:{\upbeta}$^70,71^. Inhibition of this pathway by PGV-5 and HGV-5 can reduce downstream regulation involved in tumor growth, malignant process, and resistance in cancer therapy.

Nuclear Factor Kappa B Subunit 1 (NF-κB1) signaling pathway is crucial for regulating immune response, inflammation, and cell survival. It activates several key components, including PLC$\:{\upgamma\:}$ (Phospholipase C Gamma), PKC (Protein Kinase C), and IKK (IκB Kinase). PLC$\:{\upgamma\:}$ activity produced DAG which activates PKC and phosphorylates IKK. This phosphorylation is essential for the degradation of IκB protein, triggering transcription of target genes involved in inflammation, immunity, and cell survival^72^.

The Signal Transducer and Activator of Transcription 3 (STAT3) pathway is crucial in cancer biology, affecting activities like cell proliferation, survival, migration, and immune evasion. Aberrant activation of STAT3 is common in malignancies, facilitating tumor development and metastasis. It is activated through the JAK/STAT signaling pathway, wherein cytokines or growth factors (e.g., IL-6 and EGF) stimulate receptor-associated kinases (JAKs) to phosphorylate STAT3 via the SH2 domain. STA3 dimers and translocates to the nucleus, interacting with upstream promoter regions of target genes to initiate transcription. This process results in tumor proliferation and malignant advancement of cancer^73–75^. Various inhibitors have been developed, such as PGV-5 and HGV-5 which can reduce the expression of genes supporting proliferation, tumor vasculature formation, and suppress tumor growth.

P-gp upregulation due to AKT, STAT3, EGFR, and NF-κB1 activation leads to increased efflux of chemotherapeutic agents from cancer cells, contributing to acquired drug resistance. The interplay between these signaling and P-gp expression creates a feedback loop that can perpetuate resistance mechanism. Inhibiting these signaling pathways or its downstream effects may provide a strategy to reduce P-gp levels and enhance the efficacy of chemotherapy and reversing MDR by decreasing P-gp expression^76^.

A study combining comprehensive in silico studies and acute toxicity, has shown that these methods can be effective in drug discovery but has limitations. It can inaccurately represent the complexity of biological systems, leading to oversimplified models and focus on short-term exposure only, which may not accurately reflect long term effects of chronic exposure. PGV-5 and HGV-5 exhibit promising toxicity profiles, suggesting that formulation modifications may improve efficacy and safety. They also showed superior result in in silico analyses compared to curcumin, indicating potential for further development. Additional experiments are required to explore their therapeutic potential.

## Conclusion

The study explores the potential of PGV-5 and HGV-5 in addressing multidrug resistance in cancer. PGV-5, classified as GHS class 4 due to lung morphology changes, and HGV-5, classified as GHS class 5 due to alterations in heart morphology. Both compounds exhibit a favorable ADME profile, suggesting their suitability for further preclinical development, particularly due to their inhibitory effects on P-glycoprotein (P-gp). Findings from interaction studies, docking affinity scores, and molecular dynamics simulations of the P-gp receptor indicate that structural modifications of curcumin—specifically, the addition of a methoxy group and the conversion of the diketone backbone to a monoketone, as seen in PGV-5 and HGV-5—are highly likely to enhance anti-MDR activity through P-gp modulation. The established pharmacodynamic mechanism of curcumin in MDR inhibition primarily involves the PI3K/Akt/NF-κB signaling pathway. Additionally, bioinformatics analysis via network pharmacology mapping further substantiates the anticancer potential of PGV-5 and HGV-5, revealing a complex mechanism of action that closely parallels curcumin’s role in suppressing cancer progression and enhancing antitumor immune responses. These effects are mediated through key molecular targets, including AKT1, STAT3, EGFR, and NF-κB1. Despite their therapeutic potential, the toxicity profiles of PGV-5 and HGV-5 present a challenge. To mitigate these toxic effects, various strategies such as formulation optimization, dose adjustment, targeted delivery, and combination therapy may be employed. Further studies are required to elucidate post-treatment molecular expression profiles in cancer cells.

## Electronic supplementary material

Below is the link to the electronic supplementary material.

Supplementary Material 1

## Data Availability

All data generated or analysed during this study are included in this published article [and its supplementary information files].

## References

[1] Szakács, G., Paterson, J. K., Ludwig, J. A., Booth-Genthe, C. & Gottesman, M. M. Targeting multidrug resistance in cancer. *Nat. Rev. Drug Discov.* **5**, 219–234 (2006). 16518375 10.1038/nrd1984

[2] Liscovitch, M. & Lavie, Y. Cancer multidrug resistance: A review of recent drug discovery research. *IDrugs* **5**, 349–355 (2002). 15565517

[3] Eckford, P. D. W. & Sharom, F. J. ABC efflux pump-based resistance to chemotherapy drugs. *Chem. Rev.* **109**, 2989–3011 (2009). 19583429 10.1021/cr9000226

[4] Krishna, R. & Mayer, L. D. Multidrug resistance (MDR) in cancer. *Eur. J. Pharm. Sci.* **11**, 265–283 (2000). 11033070 10.1016/s0928-0987(00)00114-7

[5] Palmeira, A. et al. Dual inhibitors of P-glycoprotein and tumor cell growth: (Re)discovering thioxanthones. *Biochem. Pharmacol.* **83**, 57–68 (2012). 22044878 10.1016/j.bcp.2011.10.004

[6] Itokawa, H., Shi, Q., Akiyama, T., Morris-Natschke, S. L. & Lee, K. H. Recent advances in the investigation of curcuminoids. *Chin. Med.* **3**, 11 (2008). 18798984 10.1186/1749-8546-3-11 PMC2576304

[7] Jayaprakasha, G. K., Mohan Rao, J., Sakariah, K. K. & L. & Chemistry and biological activities of *C. longa*. *Trends Food Sci. Technol.* **16**, 533–548 (2005).

[8] Aggarwal, B. B. & Harikumar, K. B. Potential therapeutic effects of Curcumin, the anti-inflammatory agent, against neurodegenerative, cardiovascular, pulmonary, metabolic, autoimmune and neoplastic diseases. *Int. J. Biochem. Cell. Biol.* **41**, 40–59 (2009). 18662800 10.1016/j.biocel.2008.06.010 PMC2637808

[9] Anuchapreeda, S., Leechanachai, P., Smith, M. M., Ambudkar, S. V. & Limtrakul, P. Modulation of P-glycoprotein expression and function by Curcumin in multidrug-resistant human KB cells. *Biochem. Pharmacol.* **64**, 573–582 (2002). 12167476 10.1016/s0006-2952(02)01224-8

[10] Chearwae, W., Anuchapreeda, S., Nandigama, K., Ambudkar, S. V. & Limtrakul, P. Biochemical mechanism of modulation of human P-glycoprotein (ABCB1) by Curcumin I, II, and III purified from turmeric powder. *Biochem. Pharmacol.* **68**, 2043–2052 (2004). 15476675 10.1016/j.bcp.2004.07.009

[11] Bonferoni, M. C., Rossi, S., Sandri, G. & Ferrari, F. Nanoparticle formulations to enhance tumor targeting of poorly soluble polyphenols with potential anticancer properties. *Semin. Cancer Biol.* **46**, 205–214 (2017). 28673607 10.1016/j.semcancer.2017.06.010

[12] Li, Z. et al. Pluronics modified liposomes for Curcumin encapsulation: Sustained release, stability and bioaccessibility. *Food Res. Int.* **108**, 246–253 (2018). 29735054 10.1016/j.foodres.2018.03.048

[13] Anand, P., Kunnumakkara, A. B., Newman, R. A. & Aggarwal, B. B. Bioavailability of Curcumin: Problems and promises. *Mol. Pharm.* **4**, 807–818 (2007). 17999464 10.1021/mp700113r

[14] Um, Y. et al. Synthesis of Curcumin mimics with multidrug resistance reversal activities. *Bioorg. Med. Chem.* **16**, 3608–3615 (2008). 18295490 10.1016/j.bmc.2008.02.012

[15] Revalde, J. L., Li, Y., Hawkins, B. C., Rosengren, R. J. & Paxton, J. W. Heterocyclic cyclohexanone monocarbonyl analogs of Curcumin can inhibit the activity of ATP-binding cassette transporters in cancer multidrug resistance. *Biochem. Pharmacol.* **93**, 305–317 (2015). 25543853 10.1016/j.bcp.2014.12.012

[16] Reksohadiprodjo, M. S. et al. Derivatives of benzylidene cyclohexanone, benzylidene cyclopentanone, and benzylidene acetone, and therapeutic uses thereof. (2004).

[17] Eryanti, Y. et al. Synthesis, structure-activity relationship, Docking and molecular dynamic simulation of Curcumin analogues against HL-60 for anti cancer agents (Leukemia). *Orient. J. Chem.* **33**, 2164–2172 (2017).

[18] Fu, L. et al. ADMETlab 3.0: An updated comprehensive online ADMET prediction platform enhanced with broader coverage, improved performance, API functionality and decision support. *Nucleic Acids Res.* **52**, W422–W431 (2024). 38572755 10.1093/nar/gkae236 PMC11223840

[19] Lubis, A., Wardhani, F. M., Lubis, A. T. & Tandanu, E. Cerebral histopathology in acute toxicity test of *Curcuma Zedoria*. *Jurnal Penelitian Pendidikan IPA* **9**, 8368–8375 (2023).

[20] Vilar, S., Sobarzo-Sánchez, E. & Uriarte, E. In Silico prediction of p-glycoprotein binding: Insights from molecular Docking studies. *Curr. Med. Chem.* **26**, 1746–1760 (2019). 29189117 10.2174/0929867325666171129121924

[21] *Test No. 420: Acute Oral Toxicity - Fixed Dose Procedure*. OECD, (2002). 10.1787/9789264070943-en

[22] Vilar, S., Cozza, G. & Moro, S. Medicinal chemistry and the molecular operating environment (MOE): Application of QSAR and molecular docking to drug discovery. *Curr. Top. Med. Chem.* **8**, 1555–1572 (2008). 19075767 10.2174/156802608786786624

[23] Burley, S. K. et al. RCSB protein data bank: Biological macromolecular structures enabling research and education in fundamental biology, biomedicine, biotechnology and energy. *Nucleic Acids Res.* **47**, D464–D474 (2019). 30357411 10.1093/nar/gky1004 PMC6324064

[24] Safran, M. et al. GeneCards Version 3: The human gene integrator. *Database* baq020–baq020. (2010). 10.1093/database/baq020 PMC2938269 20689021

[25] Daina, A., Michielin, O., Zoete, V. & SwissTargetPrediction Updated data and new features for efficient prediction of protein targets of small molecules. *Nucleic Acids Res.* **47**, W357–W364 (2019). 31106366 10.1093/nar/gkz382 PMC6602486

[26] Gallo, K., Goede, A., Preissner, R. & Gohlke, B. O. SuperPred 3.0: Drug classification and target prediction—A machine learning approach. *Nucleic Acids Res.* **50**, W726–W731 (2022). 35524552 10.1093/nar/gkac297 PMC9252837

[27] Heberle, H. et al. A web-based tool for the analysis of sets through Venn diagrams. *BMC Bioinform.* **16**, 169 (2015). 10.1186/s12859-015-0611-3 PMC4455604 25994840

[28] Szklarczyk, D. et al. The STRING database in 2023: Protein–protein association networks and functional enrichment analyses for any sequenced genome of interest. *Nucleic Acids Res.* **51**, D638–D646 (2023). 36370105 10.1093/nar/gkac1000 PMC9825434

[29] Shannon, P. et al. Cytoscape: A software environment for integrated models of biomolecular interaction networks. *Genome Res.* **13**, 2498–2504 (2003). 14597658 10.1101/gr.1239303 PMC403769

[30] Liao, Y., Wang, J., Jaehnig, E. J., Shi, Z. & Zhang, B. WebGestalt 2019: Gene set analysis toolkit with revamped UIs and APIs. *Nucleic Acids Res.* **47**, W199–W205 (2019). 31114916 10.1093/nar/gkz401 PMC6602449

[31] Tsaioun, K. Evidence-based absorption, distribution, metabolism, excretion (ADME) and its interplay with alternative toxicity methods. *ALTEX* 343–358. 10.14573/altex.1610101 (2016). 10.14573/altex.1610101 27806179

[32] Sareen, S., Joseph, L. & Mathew, G. Improvement in solubility of poor water-soluble drugs by solid dispersion. *Int. J. Pharm. Investig.* **2**, 12 (2012). 23071955 10.4103/2230-973X.96921 PMC3465159

[33] Gupta, D., Bhatia, D., Dave, V., Sutariya, V. & Varghese Gupta, S. Salts of therapeutic agents: Chemical, physicochemical, and biological considerations. *Molecules* **23**, 1719 (2018). 10.3390/molecules23071719 PMC6100526 30011904

[34] Bhalani, D. V., Nutan, B., Kumar, A. & Singh Chandel, A. K. Bioavailability enhancement techniques for poorly aqueous soluble drugs and therapeutics. *Biomedicines* **10**, 2055 (2022). 10.3390/biomedicines10092055 PMC9495787 36140156

[35] Lipinski, C. A. Lead- and drug-like compounds: The rule-of-five revolution. *Drug Discov. Today Technol.* **1**, 337–341 (2004). 24981612 10.1016/j.ddtec.2004.11.007

[36] Ursu, O., Rayan, A., Goldblum, A. & Oprea T. I. Understanding drug-likeness. *WIREs Comput. Mol. Sci.* **1**, 760–781 (2011).

[37] Walters, W. P. Going further than Lipinski’s rule in drug design. *Expert Opin. Drug Discov.* **7**, 99–107 (2012). 22468912 10.1517/17460441.2012.648612

[38] Johnson, T. W., Dress, K. R. & Edwards, M. Using the golden triangle to optimize clearance and oral absorption. *Bioorg. Med. Chem. Lett.* **19**, 5560–5564 (2009). 19720530 10.1016/j.bmcl.2009.08.045

[39] Kosugi, T. & Ohue, M. Quantitative estimate index for early-stage screening of compounds targeting protein-protein interactions. *Int. J. Mol. Sci.* **22**, 10925 (2021). 34681589 10.3390/ijms222010925 PMC8539639

[40] Li, B. et al. DrugMetric: Quantitative drug-likeness scoring based on chemical space distance. *Brief Bioinform.* **25** (2024). 10.1093/bib/bbae321 PMC11229036 38975893

[41] Gu, Y. et al. DBPP-predictor: A novel strategy for prediction of chemical drug-likeness based on property profiles. *J. Cheminform.* **16**, 4 (2024). 38183072 10.1186/s13321-024-00800-9 PMC10771006

[42] Ahmad, I. et al. Computational pharmacology and computational chemistry of 4-hydroxyisoleucine: Physicochemical, pharmacokinetic, and DFT-based approaches. *Front. Chem.* **11** (2023). 10.3389/fchem.2023.1145974 PMC10133580 37123881

[43] Lakey-Beitia, J., Burillo, A. M., La Penna, G., Hegde, M. L. & Rao, K. S. Polyphenols as potential metal chelation compounds against Alzheimer’s disease. *J. Alzheimer’s Dis.* **82**, S335–S357 (2021). 32568200 10.3233/JAD-200185 PMC7809605

[44] Feng, B. et al. Validation of human MDR1-MDCK and BCRP-MDCK cell lines to improve the prediction of brain penetration. *J. Pharm. Sci.* **108**, 2476–2483 (2019). 30794795 10.1016/j.xphs.2019.02.005

[45] Nanayakkara, A. K. et al. Targeted inhibitors of P-glycoprotein increase chemotherapeutic-induced mortality of multidrug resistant tumor cells. *Sci. Rep.* **8**, 967 (2018). 29343829 10.1038/s41598-018-19325-x PMC5772368

[46] Cohen, L. H. & Nicoll-Griffith, D. A. Plasma protein binding methods in drug discovery and development: Bioanalysis. in *Encyclopedia of Drug Metabolism and Interactions* 1–18 (Wiley, 2012). 10.1002/9780470921920.edm114.

[47] Liu, X., Chen, C. & Hop, E. C. A. Do we need to optimize plasma protein and tissue binding in drug discovery? *Curr. Top. Med. Chem.* **11**, 450–466 (2011). 21320069 10.2174/156802611794480918

[48] Grover, A. & Benet, L. Z. Effects of drug transporters on volume of distribution. *AAPS J.* **11**, 250–261 (2009). 19399628 10.1208/s12248-009-9102-7 PMC2691462

[49] Persidsky, Y., Ramirez, S. H., Haorah, J. & Kanmogne, G. D. Blood–brain barrier: Structural components and function under physiologic and pathologic conditions. *J. Neuroimmune Pharmacol.* **1**, 223–236 (2006). 18040800 10.1007/s11481-006-9025-3

[50] Smith, D. A., Beaumont, K., Maurer, T. S. & Di, L. Relevance of half-life in drug design. *J. Med. Chem.* **61**, 4273–4282 (2018). 29112446 10.1021/acs.jmedchem.7b00969

[51] Robinson, S. et al. A European pharmaceutical company initiative challenging the regulatory requirement for acute toxicity studies in pharmaceutical drug development. *Regul. Toxicol. Pharmacol.* **50**, 345–352 (2008). 18295384 10.1016/j.yrtph.2007.11.009

[52] Kadiyala, I. & Tan, E. Formulation approaches in mitigating toxicity of orally administrated drugs. *Pharm. Dev. Technol.* **18**, 305–312 (2013). 23317423 10.3109/10837450.2012.734516

[53] Nevskaya, A. A. et al. Nature-inspired 1-Phenylpyrrolo[2,1-a]isoquinoline scaffold for novel antiproliferative agents circumventing P-glycoprotein-dependent multidrug resistance. *Pharmaceuticals* **17**, 539 (2024). 38675499 10.3390/ph17040539 PMC11054433

[54] Jain, S., Grandits, M. & Ecker, G. F. Interspecies comparison of putative ligand binding sites of human, rat and mouse P-glycoprotein. *Eur. J. Pharm. Sci.* **122**, 134–143 (2018). 29936088 10.1016/j.ejps.2018.06.022 PMC6422297

[55] Syed, S. B. et al. Targeting P-glycoprotein: Investigation of Piperine analogs for overcoming drug resistance in cancer. *Sci. Rep.* **7**, 7972 (2017). 28801675 10.1038/s41598-017-08062-2 PMC5554262

[56] Kumbhar, N., Khan, N., Bavi, R., Barage, S. & Khan, A. Reversal of P-glycoprotein mediated multidrug resistance in MCF-7/R cancer cells by Esculetin derivatives: Experimental and MD simulation studies. *Am. J. Biomed. Life Sci.* **12**, 30–48 (2024).

[57] Tang, X., Cao, J., Bi, H. & Feng, J. Effect of Curcumin on multidrug resistance in resistant human gastric carcinoma cell line SGC7901/VCR. *Acta Pharmacol. Sin.* **26**, 1009–1016 (2005). 10.1111/j.1745-7254.2005.00149.x 16038636

[58] Lu, W. D., Qin, Y., Yang, C., Li, L. & Fu, Z. X. Effect of Curcumin on human colon cancer multidrug resistance in vitro and in vivo. *Clinics* **68**, 694–701 (2013). 23778405 10.6061/clinics/2013(05)18 PMC3654338

[59] Choi, B. H., Kim, C. G., Lim, Y., Shin, S. Y. & Lee, Y. H. Curcumin down-regulates the multidrug-resistance mdr1b gene by inhibiting the PI3K/Akt/NFκB pathway. *Cancer Lett.* **259**, 111–118 (2008). 18006147 10.1016/j.canlet.2007.10.003

[60] Follit, C. A., Brewer, F. K., Wise, J. G. & Vogel, P. D. In Silico identified targeted inhibitors of P-glycoprotein overcome multidrug resistance in human cancer cells in culture. *Pharmacol. Res. Perspect.* **3**, (2015). 10.1002/prp2.170 PMC4618641 26516582

[61] Li, L. et al. Network pharmacology: A bright guiding light on the way to explore the personalized precise medication of traditional Chinese medicine. *Chin. Med.* **18**, 146 (2023). 37941061 10.1186/s13020-023-00853-2 PMC10631104

[62] Peng, Y. S. et al. Incidence and relative risk for developing cancers in women with gestational diabetes mellitus: A nationwide cohort study in Taiwan. *BMJ Open.* **9**, e024583 (2019). 30796123 10.1136/bmjopen-2018-024583 PMC6398720

[63] Slouha, E. et al. The relationship between gestational diabetes and the risk of cancer: A systematic review. *Cureus*10.7759/cureus.53328 (2024). 38435884 10.7759/cureus.53328 PMC10906975

[64] Cha, J. H., Chan, L. C., Li, C. W., Hsu, J. L. & Hung, M. C. Mechanisms controlling PD-L1 expression in cancer. *Mol. Cell.* **76**, 359–370 (2019). 31668929 10.1016/j.molcel.2019.09.030 PMC6981282

[65] Zhou, Y. J. et al. PD-L1: Expression regulation. *Blood Sci.* **5**, 77–91 (2023). 37228770 10.1097/BS9.0000000000000149 PMC10205351

[66] Wee, P. & Wang, Z. Epidermal growth factor receptor cell proliferation signaling pathways. *Cancers (Basel)* **9**, 52 (2017). 28513565 10.3390/cancers9050052 PMC5447962

[67] Uribe, M. L., Marrocco, I. & Yarden, Y. EGFR in cancer: Signaling mechanisms, drugs, and acquired resistance. *Cancers (Basel)* **13**, 2748 (2021). 34206026 10.3390/cancers13112748 PMC8197917

[68] Plattner, C. & Hackl, H. Modeling therapy resistance via the < scp > egfr signaling pathway. *FEBS J.* **286**, 1284–1286 (2019). 30892828 10.1111/febs.14809 PMC6850018

[69] Ali, R., Brown, W., Purdy, S. C., Davisson, V. J. & Wendt, M. K. Biased signaling downstream of epidermal growth factor receptor regulates proliferative versus apoptotic response to ligand. *Cell. Death Dis.* **9**, 976 (2018). 30250119 10.1038/s41419-018-1034-7 PMC6155319

[70] Kaidanovich-Beilin, O. & Woodgett, J. R. GSK-3: Functional insights from cell biology and animal models. *Front Mol. Neurosci.* **4** (2011). 10.3389/fnmol.2011.00040 PMC3217193 22110425

[71] van der Vos, K. E. & Coffer, P. J. The extending network of FOXO transcriptional target genes. *Antioxid. Redox Signal.* **14**, 579–592 (2011). 20673124 10.1089/ars.2010.3419

[72] Napetschnig, J. & Wu, H. Molecular basis of NF-κB signaling. *Annu. Rev. Biophys.* **42**, 443–468 (2013). 23495970 10.1146/annurev-biophys-083012-130338 PMC3678348

[73] Mohan, C. D. et al. Targeting STAT3 signaling pathway in cancer by agents derived from mother nature. *Semin Cancer Biol.* **80**, 157–182 (2022). 32325172 10.1016/j.semcancer.2020.03.016

[74] Chang, N. S., To, K. K., Liou, Y. C., Li, Y. J. & Editorial The role of STAT3 signaling pathway in tumor progression. *Front. Oncol.* **13**, (2023). 10.3389/fonc.2023.1151862 PMC9983217 36874126

[75] Wingelhofer, B. et al. Implications of STAT3 and STAT5 signaling on gene regulation and chromatin remodeling in hematopoietic cancer. *Leukemia* **32**, 1713–1726 (2018). 29728695 10.1038/s41375-018-0117-x PMC6087715

[76] Seelig, A. P-glycoprotein: One mechanism, many tasks and the consequences for pharmacotherapy of cancers. *Front. Oncol.* **10** (2020). 10.3389/fonc.2020.576559 PMC7649427 33194688

